# Phylogenetic Authentication of Amplicon Sequence Variants in Single‐Specimen Metabarcoding of Tropical Insects

**DOI:** 10.1111/1755-0998.70178

**Published:** 2026-07-08

**Authors:** Sarawut Ounjai, Huaxi Liu, Zichen Zhou, Maria Pestana Correia, Thomas J. Creedy, Carmelo Andújar, Paula Arribas, Alfried P. Vogler

**Affiliations:** ^1^ Department of Life Sciences Natural History Museum London UK; ^2^ Department of Life Science Imperial College London Ascot UK; ^3^ Centre for Environmental Policy Imperial College London London UK; ^4^ Instituto de Productos Naturales y Agrobiología (IPNA–CSIC) San Cristóbal de La Laguna Tenerife Spain

**Keywords:** ASV authentication, biodiversity assessment, mitochondrial metagenomics, molecular taxonomy, phylogenetic placement, tropical diversity

## Abstract

High‐throughput sequencing (HTS) allows large‐scale DNA barcoding of individually tagged specimens (‘megabarcoding’), but deep amplicon sequencing produces a mixture of authentic mitochondrial sequences together with nuclear pseudogenes (NUMTs), environmental and cross‐sample contaminants, and sequencing artefacts. Standard approaches relying on read clustering or dominant‐read selection often fail to classify these types, leading to incorrect taxonomic identifications and species counts. We developed an authentication framework by integrating abundance filtering, phylogenetic placement and taxonomic congruence. The workflow was applied to 18,533 morphospecies of tropical beetles (Coleoptera) from multiple biogeographic regions, which were imaged for family‐level identification, prior to individual Illumina barcoding. Sequencing yielded > 36 million reads and 64,544 unique ASVs, which were evaluated against a reference phylogeny of > 13,000 mitogenomes. Authentication succeeded for 86.5% of quality‐passing specimens (15,901 ASVs). Non‐authentic sequences were technical artefacts (58.0%), environmental contamination including prey DNA (14.2%), intra‐individual variants (NUMTs, heteroplasmy; 11.3%) and cross‐sample contamination (7.5%). Authentication success and the proportions of failure categories varied markedly across trap types, sampling campaigns, taxonomic groups and sequencing runs. We identified 930 confirmed NUMTs based on consistent co‐occurrence patterns and phylogenetic proximity to authenticated haplotypes. Single‐specimen HTS data contain substantial biological and technical complexity not resolved by standard filtering methods. Our pipeline‐agnostic, phylogenetically informed authentication framework achieves robust recovery of validated barcodes while retaining informative secondary variants, improving the accuracy of molecular ASV data to a standard sufficient for inclusion in barcode reference databases and the phylogenetically informed DNA barcoding of tropical insects.

## 1 Introduction

DNA barcoding has been transformed by high‐throughput sequencing (HTS) technologies, which enable the analysis of PCR amplicon mixtures from multiple specimens, replacing traditional Sanger sequencing limited to single templates (Hebert et al. 2003; Taberlet et al. 2012). While HTS of amplicons is widely adopted for metabarcoding of ecological communities, it is also increasingly used for genotyping of individual specimens, sometimes termed ‘megabarcoding’ (Chua et al. 2023). Using the mitochondrial COI marker, single‐specimen tagging on HTS platforms enables the multiplexing of thousands (Srivathsan et al. 2019) or even 100,000 (Hebert et al. 2025) specimens in a single sequencing run. The addition of sequence tags during PCR, multiplied by tags in the library construction, has thus become a cost‐effective method for large‐scale DNA‐based species identification in complex samples of invertebrates (Chua et al. 2023; Liu et al. 2017; Meier et al. 2016).

HTS (meta)barcoding data are generally analysed by grouping sequence reads into Operational Taxonomic Units (OTUs) based on nucleotide similarity, using a variety of clustering methods (Mahé et al. 2022; Rognes et al. 2016; Edgar 2010; Schloss et al. 2009). This approach accommodates the variation of amplicons usually ascribed to sequencing or PCR errors that survived the various filtering procedures applied to the raw data. However, as platform accuracy has improved especially for Illumina‐type sequencers, reads can now be resolved at the level of individual genotypes as Amplicon Sequence Variants (ASVs) that presumably represent the true DNA templates in a sample (Callahan et al. 2017). Deep amplicon sequencing on Illumina platforms also recovers a wide range of variants of different origins, including traces of non‐targets (e.g., prey, symbionts or parasites), low‐copy nuclear pseudogenes (‘nuclear mitochondrial’, NUMTs) and heteroplasmic mitochondrial variants (Song et al. 2008). In addition, amplification may be affected by cross‐contamination among samples, in particular when mixtures from bulk traps affected by varying levels of degradation are sequenced in the same run. In total, deep sequencing of amplicons may yield surprisingly large numbers of extraneous sequence reads, whose combined count may even exceed that of the target haplotype, as observed in Illumina barcoding of single bee specimens (Creedy et al. 2020).

Most ‘megabarcoding’ studies still rely on read clustering, in part necessitated by the use of higher‐error Oxford Nanopore Technology (ONT) platforms, which require the assembly of a consensus from multiple reads and thus preclude strict ASV calling (Hebert et al. 2025; Srivathsan et al. 2024). This approach is partly driven by the scientific objectives of such studies, which focus on high specimen throughput, for example, for community‐level abundance studies, and matching to known reference sequences (Hebert et al. 2025). However, the methodology is less powerful for applications requiring exact sequence reads, for example, those focused on genetic variation in community genetics (Noguerales et al. 2023) or the generation of new reference sequences for addition to the growing barcode databases (Srivathsan et al. 2024). Finally, even when clustering is used, the presence of multiple distantly related sequences in an amplicon mixture requires decisions about filtering strategies, which are either not reported (Jabot et al. 2025; Srivathsan et al. 2021) or based simply on the top‐abundant variant (Hebert et al. 2025; Meier et al. 2016). Relying solely on the dominant mitochondrial haplotypes may overlook potential cross‐contamination of specimens in the trap, the laboratory or in the library tagging step. In addition, removing these sequences from consideration also represents a loss of valuable biological information, for example, for studying trophic interactions and ecological associations.

The prevalence of such secondary reads varies among samples, depending on multiple factors that influence ASV diversity and error rates. DNA degradation is common in biodiversity samples used for barcoding and metabarcoding, particularly from tropical field sites gathered under suboptimal environmental conditions, which can compromise DNA quality, increase PCR error rates, bias the proportions of authentic mitochondrial variants, and increase the risk of cross–contamination in the most highly degraded samples. The impact of NUMTs is similarly unpredictable, as whole‐genome studies have shown uneven distribution across taxa and chromosomes, yet these effects still need to be studied across a wider spectrum of lineages (He et al. 2025; Hebert et al. 2023). Compounding these issues are the limited taxonomic knowledge and lack of reference coverage for most insect groups, especially in tropical regions, which hamper both the species identification via database matches and the discrimination of authentic versus non‐target sequences (Andújar et al. 2021; Souto‐Vilarós et al. 2025; Zinger et al. 2025).

To address these issues, we developed an analytical framework that distinguishes authentic mitochondrial sequences from nuclear pseudogenes, technical artefacts and contamination, while producing accurate taxonomic assignments even when the coverage of the reference sequence is limited. Our workflow combines the widely applied abundance–based criteria for selecting authentic ASVs with a phylogenetic characterisation of ASVs as either distantly related non‐targets (e.g., ingested prey and internal parasites) or closely related NUMTs. The approach requires a phylogenetic tree against which ASVs are assessed, and it was applied here to a set of > 18,000 beetle (Coleoptera) specimens, each thought to represent a different morphospecies from poorly characterised tropical forest communities, which were amplicon sequenced with individual tags on an Illumina platform. The authentication of reads required the correct assignment of ASVs at the family level. There are some 200 families in the Coleoptera, most of which can be recognised from specimen images and thus can be compared to the phylogenetic position of corresponding sequence reads. We assembled a densely sampled tree from full‐length mitogenome sequences of Coleoptera (Carpenter and Vogler 2025; Creedy, Ding, et al. 2026; Creedy, Lee, et al. 2026), which was used to characterise the ASV mixtures obtained for each specimen and to determine authentic mitochondrial haplotypes. The success of ASV authentication was compared across different sample batches, trap types, preservation conditions, biogeographic realms and major lineages of Coleoptera. The results highlight the need for stringent characterisation of single‐specimen HTS barcode mixtures, especially if they are used as de novo‐generated reference sequences missing from existing reference databases.

## 2 Methods

**FIGURE 1:**
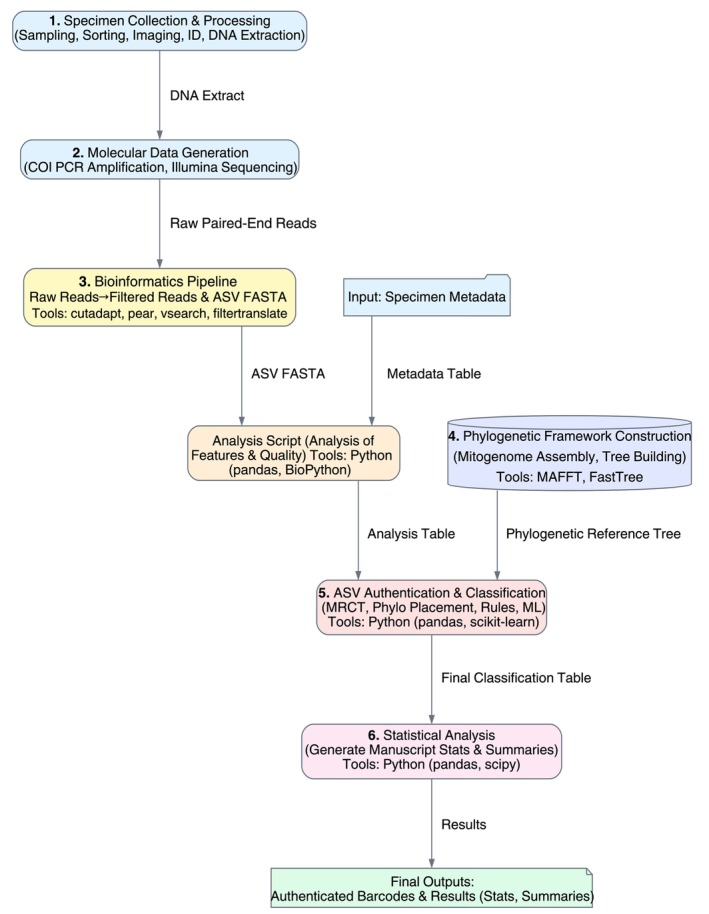
Overview of the integrated ASV analysis workflow. The diagram illustrates the main stages described in Sections 2.1, 2.6, from (1) specimen collection and processing and (2) molecular data generation to (3) the bioinformatics pipeline for ASV calling, alongside (4) phylogenetic framework construction to build a reference tree. The core of the workflow is (5) ASV authentication and classification, which integrates ASV data and phylogenetic placement, followed by (6) statistical analysis to generate final authenticated barcodes and summaries.

The workflow for generating authenticated COI barcodes from mass‐trapped tropical insects comprised a multi‐step protocol from wet‐laboratory amplicon generation to ASV selection, ultimately characterising all ASVs as either authentic mitochondrial barcodes for taxonomic assignment or other types of ASVs representing secondary variants (NUMTs/heteroplasmy), external DNA, cross‐sample contaminants and technical artefacts (Figure 1).

### 2.1 Specimen Collection and Processing

Beetle specimens were collected across 14 countries in four zoogeographic regions: Neotropics (Ecuador, Mexico, Honduras, Panama, French Guiana), Indo–Malaya (Malaysia, Thailand, China, India), Afrotropics (Equatorial Guinea, Mozambique, South Africa) and Palearctic (United Kingdom, Palestine). Specimens were obtained using 11 different sampling methods, comprising both passive and active approaches. Passive methods included flight interception traps, Malaise traps, pitfall traps, pan traps, Winkler extractors for leaf litter and SLAM traps. Active collection methods comprised hand collecting, canopy fogging, sweep netting and light‐trapping. Traps were typically exposed for up to 1 week between collection events, using either 96% ethanol or SDS + EDTA buffer as preservation fluid. Samples were transported to the laboratory under ambient conditions and subsequently preserved at −20°C or −80°C for long‐term storage. Detailed specimen metadata, collection protocols and taxonomic determinations are available in the project repository (https://github.com/OunjaiS/ASV‐Selection‐Taxonomic‐Assignment).

All Coleoptera specimens were sorted from trap catches and photographed in bulk using an AxioZoom large‐scale high‐resolution camera. Images were used to detect all distinct morphospecies, and a specimen image representative of each morphospecies was cropped and archived in a public repository (https://www.flickr.com/photos/site‐100/). Each specimen was identified to the family or lower level, where possible, aided by taxonomists via iNaturalist (https://www.inaturalist.org/people/site_100). DNA extractions were performed using a QIAGEN Blood and Tissue Kit (QIAGEN 2020). DNA concentrations were determined using a Qubit fluorometer (Thermo Fisher Scientific 2015), and DNA degradation was assessed via agarose gel electrophoresis.

All fieldwork and specimen collection complied with institutional and national regulations under appropriate research and export permits issued by each country's authorities (see Data Availability Statement).

### 2.2 Molecular Data Generation

A 418 bp COI fragment was amplified using tagged degenerate primers targeting a conserved region of the mitochondrial COI gene: forward (B) 5′‐CCNGAYATRGCNTTYCCNCG‐3′ (Hajibabaei et al. 2012) and reverse (FoldR) 5′‐TANACYTCNGGRTGNCCRAARAAYCA‐3′. The reverse primer is a degenerate variant of jgHCO2198 (Geller et al. 2013), with inosine bases replaced by fully degenerate positions, as used in Arribas et al. (2016). This primer pair targets a conserved region of the mitochondrial COI gene and was selected for its broad amplification success across Coleoptera, as validated in prior studies on diverse arthropod assemblages (Arribas et al. 2016; Creedy et al. 2020). Unique paired‐tag combinations enabled specimen‐specific identification during multiplexed sequencing (Data S1). PCR reactions were conducted in 25 μL volumes with the following profile: 95°C for 4 min, followed by 35 cycles of 95°C for 30 s, 48°C for 30 s and 72°C for 1 min, with a final extension at 72°C for 10 min. Products were visualised by gel electrophoresis. Amplicons were pooled in equimolar ratios and subjected to library preparation before sequencing on an Illumina MiSeq platform using 2 × 300 paired‐end chemistry across 10 independent runs conducted between 2016 and 2022.

### 2.3 Bioinformatics Pipeline

Raw sequences were demultiplexed and adapter‐trimmed with Cutadapt v3.5 (Martin 2011), and paired‐end reads were merged with PEAR v0.9.6 (Zhang et al. 2014). Quality filtering was performed using VSEARCH v2.21.1 (Rognes et al. 2016) with maximum expected error thresholds of 1.0 (‐‐fastq_maxee 1). Sequences were dereplicated using ‘vsearch ‐‐derep_fulllength’ and denoised with ‘vsearch ‐‐cluster_unoise’ to eliminate sequencing errors. Subsequent filtering steps included: (1) length filtering to retain 418 ± 3 base pair sequences, (2) translation filtering to remove sequences with stop codons or frameshifts, and (3) chimaera detection using UCHIME3 (Edgar 2016) in de novo mode. The resulting amplicon sequence variants (ASVs) were each assigned a unique identifier (Data S2). No initial taxonomic filtering was applied at this stage to avoid excluding potentially novel or divergent Coleoptera sequences. Read counts were recorded for each ASV in a single sample and across all samples and sequence runs. Taxonomic assessment and filtering of non‐Coleoptera sequences were subsequently performed through phylogenetic analysis (Section 2.4).

### 2.4 Phylogenetic Framework Construction

A phylogenetic reference tree was constructed from 13,380 complete or partial mitochondrial genome sequences. Mitogenomes were assembled from shotgun sequencing data obtained from a subset of the specimens included in the current study (*n* = 9027) published elsewhere (Carpenter and Vogler 2025; Creedy, Ding, et al. 2026; Creedy, Lee, et al. 2026). These mitogenomes were supplemented with existing Coleoptera sequences downloaded from GenBank (accessed January 2023). All mitochondrial genome sequences are provided in Data S3. Thirteen protein‐coding genes were extracted using extract_genes.py (https://github.com/tjcreedy/biotools), translated using translate.py (same repository) and aligned at the amino acid level using MAFFT v7.490 (Katoh and Standley 2013) with the G‐INS‐i algorithm and 1000 iterations. Alignments were back‐translated to nucleotide space using backtranslate.py (same repository) and concatenated using catfasta2phyml.pl (https://github.com/nylander/catfasta2phyml). The resulting supermatrix, approximately 18,600 bp in length, was used for phylogenetic analysis under maximum likelihood using FastTree v2.1.11 (Price et al. 2010) with the GTR + CAT approximation model. The resulting tree served as a backbone constraint for phylogenetic analysis of barcodes.

Three reference tree variants were constructed to assess the influence of taxon sampling density on phylogenetic position stability (Data S4): a 5k tree (5000 mitogenomes selected to maximise family‐level diversity), a 12k tree (12,000 mitogenomes combining taxonomic and phylogenetic diversity) and a 13.3k tree (complete dataset). All trees were inferred using identical FastTree methods, ensuring that topological differences reflected taxon sampling rather than methodological variation.

### 2.5 ASV Authentication and Classification Protocol

A hybrid classification system was developed by integrating abundance‐based filtering, phylogenetic validation and taxonomic congruence assessment to categorise all ASV records into five classes: authenticated mitochondrial haplotype, cross‐contamination, intra‐individual variant, environmental contamination and technical artefact.

#### 2.5.1 Minimum Read Count Threshold Determination

Minimum read count thresholds (MRCT) ranging from 1 to 20 reads per ASV were evaluated to identify the optimal balance between sequence reliability and specimen retention. For each threshold level, four metrics were calculated: (1) the proportion of specimens retaining at least one quality‐passing ASV (specimen retention rate); (2) the percentage of total ASV records retained; (3) the percentage of unique sequence variants retained; and (4) the authentication success rate (proportion of specimens that passed the threshold and yielded an authenticated ASV). The preferred MRCT maximised specimen retention whilst minimising inclusion of low‐abundance artefacts. ASVs falling below the selected threshold were excluded from authentication analyses but retained for comprehensive characterisation of secondary sequence patterns.

#### 2.5.2 Phylogenetic Analysis and Taxonomic Validation

ASVs meeting the MRCT were aligned to the COI reference region using MAFFT (L–INS–i algorithm) and subjected to phylogenetic tree searches with FastTree, employing the three reference tree variants (5k, 12k and 13.3k trees; see Section 2.4) as backbone constraints. For each ASV in each tree, two metrics were calculated: (1) the phylogenetic distance to the nearest authenticated reference sequence (cophenetic distance of query and reference under the GTR + CAT model) using the *ape* package v5.6‐2 (Paradis and Schliep 2019), and (2) the congruence between family‐level placement on the tree and the morphotaxonomic identification of the sequenced specimen. A ‘match’ status (denoted taxonomy_match = ‘match’) was assigned when phylogenetic and morphological family assignments were concordant, serving as a prerequisite for positive ASV authentication. Conversely, a non‐match status (taxonomy_match ≠ ‘match’) indicated non‐authentic ASV status. This binary variable served as a primary criterion for subsequent ASV status classification.

For beetle families absent from the mitogenome reference database, phylogenetic placement was assessed at broader taxonomic levels (superfamily or suborder), with authentication relying more heavily on abundance criteria and comparative phylogenetic distances to taxonomically proximate families. Such cases were flagged for manual review.

#### 2.5.3 Classification Framework

Once their phylogenetic position was known, ASVs were classified based on combined criteria of abundance, phylogenetic position and co‐occurrence with closely related reads (Table 1). Classification was applied at two levels: specimen‐level (authenticated, failed) and ASV‐level (authenticated, cross‐contamination, intra‐individual variant, environmental contamination, technical artefacts).

**TABLE 1:** ASV classification criteria framework.

Criterion	Authenticated ASVs	Intra‐individual variants (NUMTs/heteroplasmy)	Cross‐contamination	Environmental contamination	Technical artefacts
Read abundance	Read count above MRCT; highest abundance in sample	Meets MRCT; not highest abundance	Meets MRCT; moderate to low abundance	Meets MRCT	Below MRCT threshold (< 4 reads) OR meets MRCT but has other disqualifying properties
Phylogenetic distance from known reference	Minimal distance to reference	Low distance to reference	Not primary criterion; distance reflects source sample	Large distance to reference	Not applicable
Taxonomic placement	Matches expected family (taxonomy_match = ‘match’)	Matches expected family (taxonomy_match = ‘match’)	Irrelevant (ASV belongs to another sample's taxonomy)	Does not match expected family (taxonomy_match ≠ ‘match’)	Not applicable
Occurrence pattern	Primary ASV per specimen	Co–occurs with the same authenticated ASV in ≥ 2 specimens + similar sequence abundance ratio	Is an authenticated ASV elsewhere in the dataset	Inconsistent with the sample's expected taxonomy	Exhibits properties like abnormal length or very low sequence quality
Defining feature(s)	Highest read count + phylogenetic/morphotaxonomic match	Co‐occurrence pattern + phylogenetic/morphotaxonomic match	Sequence identity matches another sample's authenticated ASV	Phylogenetic/morphotaxonomic mismatch	Random experimental error, technical noise

‘Failed’ specimens were those that yielded no ASVs after quality filtering, possibly due to PCR inhibition, low DNA concentration, DNA degradation, primer mismatch or sequencing failure. ‘Authenticated’ ASVs were required to meet two criteria: (1) family‐level taxonomic concordance with morphological identification (taxonomy_match = ‘match’ status) in at least one of the three constraint trees, and (2) highest read abundance within the specimen's ASV pool. When multiple ASVs met these criteria, the most abundant sequence was designated the primary authenticated haplotype, with other ASVs each assigned to one of the following categories:

*Cross‐contamination* was designated when an ASV showed exact sequence identity to authenticated sequences from different specimens but was present at moderate to low abundance in the focal specimen. Contamination could occur through various pathways, including field‐based transfer (specimens in the same trap), laboratory cross‐contamination (specimens in the same extraction or PCR batch) and sequencing‐related index hopping.

*Intra‐individual variants* were characterised by strict taxonomic congruence (taxonomy_match = ‘match’ status in at least one constraint tree) and consistent co‐occurrence with other authenticated variants. Secondary ASVs detected in ≥ 2 independent specimens alongside the same authenticated ‘anchor’ ASV were classified as putative intra‐individual variants, representing nuclear (NUMTs) or heteroplasmic mitochondrial variants. The consistent co‐amplification in two or more individuals distinguished stable genomic features from sporadic technical artefacts or single‐sample contamination.

*Environmental contamination* was inferred based on cross‐family phylogenetic placement (taxonomy_match ≠ ‘match’ status across all three backbone constraint trees), indicating ASVs from taxonomically distinct organisms such as ingested prey, parasites, commensals or environmental DNA acquired during trapping and handling. In addition, ASVs with the same‐family placement but phylogenetic distances of ≥ 0.50 branch length units from all authenticated references across the three constraint trees were also considered environmental contaminants, representing divergent lineages unlikely to reflect intraspecific variation.

*Technical artefacts* included all ASVs with read counts below the MRCT threshold, encompassing sequencing errors and low‐quality amplicons. ASVs above the MRCT were also classified as artefacts if they exhibited properties inconsistent with genuine biological variants, such as abnormal sequence length or very low sequence quality. Phylogenetic validation was not applied to sequences ultimately assigned to this category.

### 2.6 Statistical Analysis

The statistical significance of authentication determinants was evaluated using chi‐square tests of independence; the Bonferroni correction was applied in Section 3.5, where each test addressed a distinct independent factor, and the Benjamini–Hochberg false discovery rate procedure was applied to the 62 codon comparisons in Section 3.6. Effect sizes were quantified using Cramér's *V* with 95% confidence intervals, interpreted as small (*V* < 0.1), medium (0.1 ≤ *V* < 0.3) or large (*V* ≥ 0.3). For continuous variables, Mann–Whitney *U* tests were used with rank‐biserial correlation (*r*) as the effect size measure.

Mutual information analysis was conducted to assess non‐linear relationships between predictor variables and authentication success. To account for the non‐independence of multiple ASVs within specimens, linear mixed models (LMMs) with specimen identity as a random intercept were fitted using lme4 (v1.1‐38; Bates et al. 2015) in R (v4.4.1); significance was assessed by likelihood‐ratio tests. Phylogenetic signal in GC content was tested using Blomberg's *K* and Pagel's *λ* (phytools v2.5‐2; Revell 2012) on an Open Tree of Life family‐level topology (rotl v3.1.1; Michonneau et al. 2016) made ultrametric by Grafen's method (ape v5.8; Paradis and Schliep 2019). All sequence‐level analyses used Python 3.11 with pandas v2.1.0, scipy v1.11.2, scikit‐learn v1.3.0, BioPython v1.81, matplotlib v3.7.2 and seaborn v0.12.2. Development and review of the Python analysis scripts were supported by Claude Code (Anthropic) (see Data Availability Statement).

## 3 Results

### 3.1 Dataset Overview and Quality Assessment

The analysis included 18,533 beetle specimens from 14 countries across four biogeographic realms, representing 119 morphologically identified families. Amplicons sequenced on Illumina MiSeq (10 runs, 2016–2022) yielded 36,459,895 reads after quality filtering, comprising 64,544 unique ASVs across 175,954 total ‘ASV records’, that is, the sum of ASVs in all samples, reflecting the presence of ASVs in multiple specimens or (in a few cases) in multiple libraries of resequenced single specimens.

**FIGURE 2:**
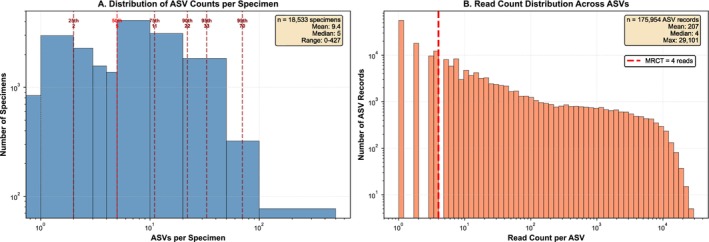
ASV complexity and read count distributions. (A) ASV per specimen distribution showing the number of specimens with a given number of ASVs (on a log scale, range 1–427), and the 25th to 95th percentiles of specimens yielding the total number of ASVs. (B) The frequency of ASVs with a certain read count. The vertical line at MRCT = 4 indicates the minimum read count threshold (MRCT); ASV records meeting or exceeding this threshold (≥ 4 reads) were retained for further analysis, whilst those below this threshold (< 4 reads) were classified as technical artefacts.

Individual specimens exhibited variable ASV complexity, with a mean of 9.5 ASVs per specimen (median: 5, range: 1–427, SD: 15.9) before abundance filtering (Figure 2A). A total of 2986 specimens (16.1%) presented a single ASV, whilst 2287 specimens (12.3%) yielded two ASVs. The 25th, 50th and 75th percentiles were 2, 5 and 11 ASVs per specimen, respectively. Read count distribution across ASV records was highly heterogeneous, with a mean of 207 reads (median: 4, range: 0–29,101, SD: 1090) (Figure 2B). The strongly right‐skewed distribution indicated that most ASV records had low abundance, with the median of four reads matching the selected minimum threshold. Quality metrics showed a mean sequence quality (*Q* score) of 35.2, with 89.3% achieving scores of *Q* > 30. Length clustering around 418 bp was tight (mean: 418.2 bp, SD: 2.1 bp), with 97.8% within 418 ± 3 bp.

The specimen set encompassed tropical rainforests (68.2%), temperate forests (18.4%), montane habitats (8.7%) and arid regions (4.7%), spanning elevations from sea level to 1200 m. Collection methods included passive traps (57.2%) and active collection (42.8%), with preservation in 96% ethanol (64.3%) or SDS + EDTA buffer (35.7%). Taxonomic representation was skewed: the five most abundant families comprised 52.1% of specimens (Staphylinidae 21.4%, Chrysomelidae 10.3%, Curculionidae 9.8%, Scarabaeidae 5.8%, Carabidae 4.8%), whilst 47 families (39.2%) had < 10 specimens each. Family‐level morphological identification confidence based on high‐resolution images was high (94.7% certain, 5.3% ambiguous).

### 3.2 Authentication Protocol and Threshold Selection

**FIGURE 3:**
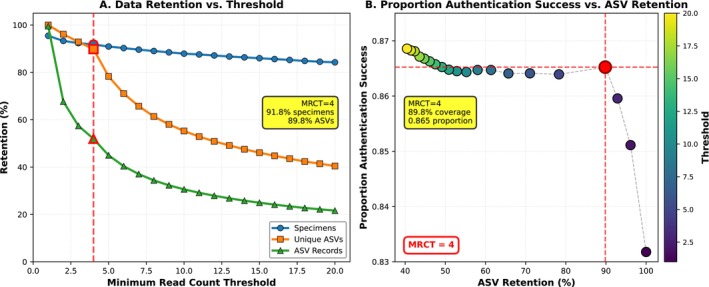
(A) The effect of varying MRCTs on data retention, separate for all ASVs, unique ASVs and specimens. (B) The correlation of authentication success and MRCT (ASV retention). Authentication success refers to the match of morphotaxonomic identification and phylogenetic placement of the corresponding ASV. The red circle marks the preferred MRCT = 4 at which point 89.8% of ASVs are retained, as is also evident from the vertical line in A.

MRCTs (1–20 reads) were systematically evaluated for the kind of ASVs removed (Figure 3A). A threshold of four reads optimally balanced coverage and reliability: it retained 17,007 specimens (91.77%) with ≥ 1 qualifying ASV, retained 91,402 ASV records (52.0%) representing 57,976 unique variants (89.8%) and achieved high authentication success rates (i.e., matching morphotaxonomic identification and phylogenetic placement at family level) that plateaued beyond this threshold (Figure 3B). The 84,552 records below the threshold (48.0%) were classified as technical artefacts (mean: 2.2 reads, median: 2.0, mode: 1.0), with 68.4% singletons and 23.7% doubletons.

Accepted specimens exhibited diverse ASV complexity, with 1–5 ASVs obtained in 52.3% of specimen‐level libraries, 6–10 ASVs in 28.1%, 11–20 ASVs in 14.2% and > 20 ASVs in 5.4% of cases (mean 5.4, median 4). This reduction from the pre‐filtering mean of 9.5 ASVs per specimen (Section 3.1) reflected the removal of low‐abundance technical artefacts through the MRCT = 4 threshold. The 1526 failed specimens (8.2%) showed non‐random distribution: failure rates varied from 4.2% to 18.7% across batches, 2.1%–24.6% across countries and 0%–89.2% across families, indicating systematic factors determining failure rates (DNA quality, amplification efficiency, batch–specific issues).

### 3.3 Phylogenetic Authentication

Phylogenetic placement within the 13,380‐mitogenome reference tree (Figure S1) authenticated 14,715 specimens (86.52% of 17,007 threshold‐passing specimens; 79.40% of the total 18,533 specimens) based on congruence with the morphological identification at family level. The remaining 2292 threshold‐passing specimens (13.48%) failed authentication despite meeting abundance criteria. The authenticated specimens were represented by 15,901 authenticated ASV records (17.40% of the total number of threshold‐passing records) representing 12,922 unique sequences.

The reference set (Section 2.4) included mitogenomes assembled from a subset of the same specimens used in this study (*n* = 9027). Of the 15,901 authenticated ASV records, 11,861 (74.6%) matched reference mitogenome sequences exactly (phylogenetic distance = 0 substitutions per site); the remaining 4040 (25.4%) were authenticated on the basis of family‐level phylogenetic congruence alone and had small but non‐zero distances to the nearest reference. In contrast, non‐authenticated sequences exhibited substantially larger distances (mean: 0.954, median: 0.315, 95th percentile: 4.34), a sharp contrast to the authenticated group (Mann–Whitney *U* test: *p* < 0.001). Authenticated ASVs also exhibited substantially elevated read counts (mean: 1904, median: 650) compared to non‐authenticated ASVs (mean: 207, median: 4); a linear mixed model confirmed that read counts were 102.7‐fold higher after accounting for between‐specimen variance (LMM: *β* = 4.63, *χ* ^2^(1) = 121,459, *p* < 2.2 × 10^−16^; ICC_specimen = 0.207; descriptive Mann–Whitney: *p* < 0.001, *r* = 0.921), confirming authenticated ASVs as the dominant mitochondrial haplotypes within each specimen. Among authenticated specimens, 13,538 (92.0%) yielded exactly one authenticated ASV, with the remainder presenting two or up to 3 ASVs (after abundance filtering).

### 3.4 Authentication Failure Analysis

**FIGURE 4:**
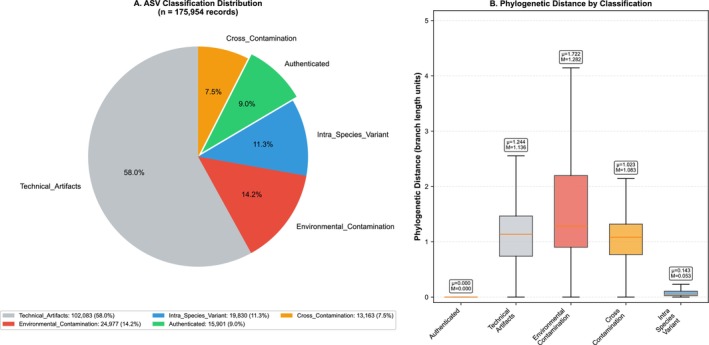
ASV classification and phylogenetic distance analysis (*n* = 175,954 records). (A) Pie chart illustrating the proportional distribution of all ASV records across the five classification categories. (B) Box plots comparing the phylogenetic distance (in branch length units) to the nearest authenticated reference for each ASV category, with mean and SD of distances shown for each group.

Of the 175,954 ASV records, 160,053 (90.96%) were not authenticated and were assigned to four failure categories (Table 2; Figure 4A), which differed systematically in phylogenetic distance, read abundance and taxonomic composition (Table 1; Figure 4B). These four classes included: (i) Technical artefacts represented the largest class, showing low mean read abundance and large phylogenetic distance to their presumed taxonomic origin. (ii) Environmental contamination displayed the highest distances, reflecting inclusion of non‐coleopteran sequences. (iii) Cross‐sample contamination exhibited intermediate distances, consistent with within–Coleoptera transfer. (iv) Secondary intra‐individual variants, which showed the lowest phylogenetic distances, with 61.7% below 0.05 branch length units, corresponding to presumed NUMTs. Several of these ASVs were encountered in more than one specimen of the same species (primary ASV), which allowed testing their association with primary authenticated ASV. Co‐occurrence analysis identified recurrent presence of 930 ASVs associated with 330 unique anchor ASVs, with 80.6% occurring in exactly two specimens, 17.1% in three to four specimens and 2.3% in five or more. These ASVs were considered to be ‘confirmed NUMTs’ representing true secondary variants consistently present in a given species, although they cannot be distinguished easily from heteroplasmic mitochondrial variants (see Section 3.6).

**TABLE 2:** Summary information for five ASV record categories (*n* = 175,954).

Category	Count	Percentage	Mean reads	Median phyl. dist.	Mean phyl. dist.	Key taxonomic/structural notes
Authenticated	15,901	9.04	1904	0	0	Taxonomic match at family level
Technical artefacts	102,083	58.02	2.20	1.136	1.244	Random distribution, no clustering
Environmental contamination	24,977	14.20	138	1.282	1.722	71.7% cross‐family Coleoptera; 19.7% non‐Coleoptera (11.4% Diptera); 2.6% bacterial
Cross‐sample contamination	13,163	7.48	100	1.083	1.023	90.0% source‐traced (78.4% lab, 15.3% field)
Intra‐individual variants	19,830	11.27	60	0.053	0.143	61.7% < 0.05 dist.; 930 confirmed NUMTs across 1078 specimens
Total	175,954	100	—	—	—	—

### 3.5 Factors Affecting Authentication Success

**FIGURE 5:**
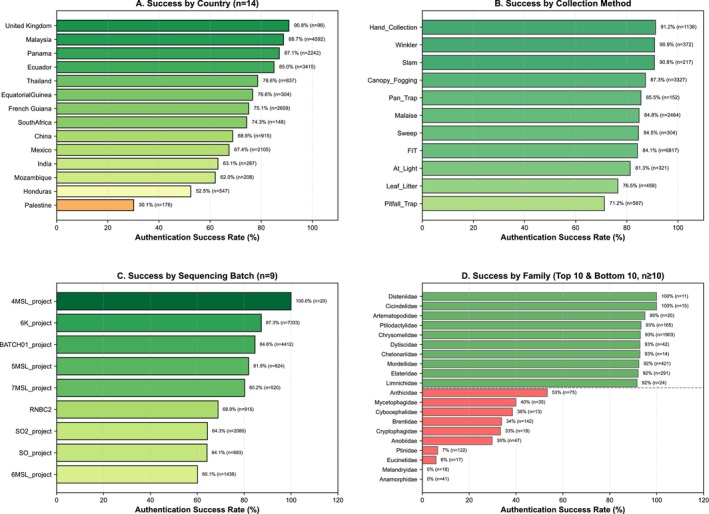
Assessment of ASV authentication success for different subsets of data, separated by: (A) geographic sets (country); (B) collection methodology; (C) sequencing batch; and (D) taxonomic family.

Authentication success varied substantially across subsets of specimens. Each of the following four factor tests addresses an independent predictor; the Bonferroni correction was applied accordingly:

1. Geographic sets (Figure 5A; Table S1) differed widely in authentication success ranging from 30.1%–90.8% (mean: 76.4%, SD: 16.2%), with the highest proportion in the United Kingdom (90.8%), Malaysia (88.7%), Panama (87.1%), and the lowest in Palestine (30.1%) and Honduras (52.5%). Within‐region variation exceeded between‐region differences, indicating that campaign‐specific effects are not affecting all specimens equally (*χ* ^2^ = 1268.25, df = 13, *p* < 0.001, Cramér's *V* = 0.262, medium effect).
2. Collection methodology (Figure 5B; Table S2) also influenced outcome (*χ* ^2^ = 563.02, df = 11, *p* < 0.001, Cramér's *V* = 0.178, medium effect). Active collection methods achieved higher success rates (Hand: 91.2%, Winkler: 90.9%, Slam: 90.8%) compared to most passive methods, though Malaise traps (84.8%) and FIT traps (84.1%) remained effective. Pitfall traps showed the lowest success (71.2%). The preservation fluid also influenced the outcomes: ethanol achieved a success rate of 88.6%, compared to 83.9% for the SDS + EDTA buffer.
3. Sequencing batch (Figure 5C; Table S3) effects were substantial (60.1%–100.0% range, mean: 81.2%, SD: 13.4%), with significant heterogeneity (*χ* ^2^ = 1181.31, df = 8, *p* < 0.001, Cramér's *V* = 0.252, medium effect). Batch success was strongly correlated with sequencing depth (*ρ* = 0.78, *p* = 0.013) and library complexity (*ρ* = 0.71, *p* = 0.032).
4. Family membership (Figure 5D, Table S4) exhibited the most substantial variation (0%–100% success range), representing the most significant effect (*χ* ^2^ = 2971.57, df = 119, *p* < 0.001, Cramér's *V* = 0.410, large effect). Among families with ≥ 10 specimens, 15 achieved a success rate of ≥ 90%: Cicindelidae and Disteniidae (both 100%), followed by Artematopodidae (95.0%), Ptilodactylidae (93.3%) and Chrysomelidae (93.0%). Conversely, 18 families exhibited < 70% success: Anamorphidae, Melandryidae (all 0.0%), Eucinetidae (5.9%), Ptinidae (6.6%). Small‐bodied families (< 3 mm) showed reduced success (52.3%) compared to medium‐bodied (3–10 mm: 81.7%) and large‐bodied (> 10 mm: 88.9%) families. Family effects remained consistent across regions (correlation *ρ* = 0.83–0.91, all *p* < 0.001), supporting a biological rather than a geographic basis.

**FIGURE 6:**
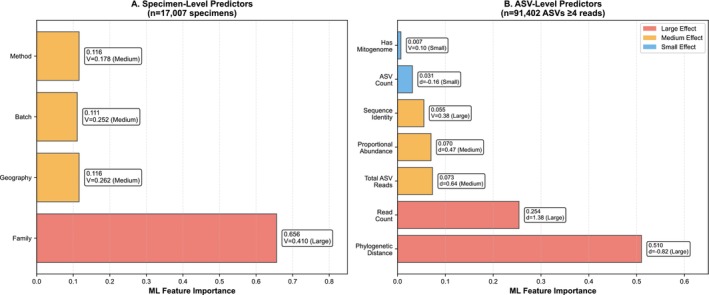
Multivariate feature importance hierarchy at specimen and ASV levels. (A) Specimen‐level predictors; (B) ASV‐level predictors.

Multivariate feature importance analysis quantified the relative contribution of each predictor of authentication success at the specimen and ASV levels (Figure 6; Table S5). At the specimen level, family identity was the strongest predictor, followed by sequencing batch, geographic sample set and collecting method. At the ASV level, phylogenetic distance to the nearest authenticated reference sequence was the dominant predictor (feature importance 0.510), reflecting the role of family‐level phylogenetic congruence as a primary criterion for authentication. Other significant predictors of authentication success included read abundance (absolute read count of the ASV; importance 0.285) and proportional representation (relative proportion of the ASV's reads within the specimen's total read pool; importance 0.142). Additional features, including total ASV count per specimen and sequence quality scores, had comparatively minor effects. Comprehensive statistical tests with effect‐size quantification for all comparisons are provided in Table S5.

### 3.6 NUMT Validation Through Codon Usage Bias

To provide molecular evidence supporting the NUMT classification (Section 3.4), codon usage and sequence composition were compared between the 930 confirmed NUMTs and their matched authenticated anchor ASVs (*n* = 330 unique ASV sequences; i.e., the specific authenticated sequences co‐occurring with confirmed NUMTs within the same specimens). We first compared GC content and codon usage within specimens using a linear mixed model (LMM: metric ~ group + (1|specimen_id)), which estimated the within‐specimen NUMT‐anchor difference after controlling for each specimen's baseline GC composition via the random intercept (*β* = fixed effect; Table S8 provides within‐specimen LMM and cross‐specimen Mann–Whitney *U* comparisons). Within‐specimen LMM revealed no significant difference in GC content, GC at any codon position or ENC between confirmed NUMTs and their matched anchor ASVs (all *p* > 0.05; Table S8; ICC_specimen = 0.896–0.949). The cross‐specimen comparison (Table S8) likewise showed no significant difference for any of these metrics (all *p* > 0.05; *r* < 0.06). The high intraclass correlations indicated that specimen identity was the dominant source of GC variance, reflecting taxon‐level GC composition rather than NUMT‐specific divergence. GC content showed no significant phylogenetic signal across 52 beetle families (Blomberg's *K* = 0.056, *p* = 0.457; Pagel's *λ* ≈ 0, *p* = 1.000), indicating the absence of any taxon‐specific effects that might affect the comparison.

**FIGURE 7:**
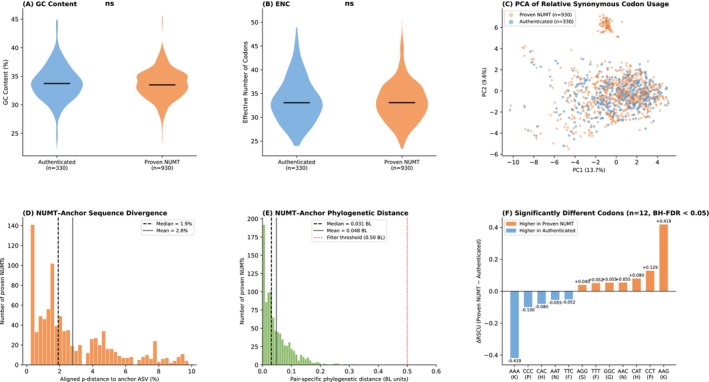
Codon usage bias and sequence divergence of confirmed NUMTs (*n* = 930) versus matched authenticated ASVs (*n* = 330; ≤ 10% p‐distance threshold). (A) GC content and (B) effective number of codons (ENC) violin plots; no significant within‐specimen difference for either metric (LMM: all *p* > 0.05; Table S8). (C) PCA of per‐ASV RSCU values; PC1 = 11.6% of variance. (D) Aligned pairwise p‐distances (Needleman–Wunsch) from each NUMT to its anchor ASV (median = 1.91%, mean = 2.79%). (E) Pair‐specific phylogenetic distances to matched anchor ASV (median = 0.031 BL, mean = 0.048 BL); dotted line = filter threshold (0.50 BL). (F) RSCU differences for the 12 significantly shifted sense codons (BH‐FDR < 0.05); ΔRSCU = Confirmed NUMT − Authenticated. Cross‐specimen comparisons in Table S8.

Relative synonymous codon usage (RSCU) analysis revealed that 12 of 62 sense codons (the Invertebrate Mitochondrial Code, transl_table = 5; NCBI 2024) differed significantly between confirmed NUMTs and their matched authenticated anchors (Benjamini–Hochberg adjusted *p* < 0.05; Table S7). Principal component analysis of per‐ASV RSCU values partially separated authenticated and confirmed NUMT clusters along PC1, which explained 11.6% of total variance (Figure 7C), indicating that while overall nucleotide composition is indistinguishable between groups, subtle differences in synonymous codon preferences persist in the 930 confirmed NUMTs relative to their anchors (Figure 7F).

Pairwise distances from each confirmed NUMT to its authenticated anchor ASV were computed using Needleman‐Wunsch global alignment as a direct measure of sequence divergence since nuclear insertion. Among 930 confirmed NUMTs, aligned pairwise distances ranged from 0.24% to 9.81% (median: 1.91%; mean: 2.79% ± 2.45%; Figure 7D), with 81.3% differing by ≤ 5% from their anchor, consistent with predominantly recent nuclear insertions. Pair‐specific phylogenetic distances from each confirmed NUMT to its matched anchor ranged from 0.001 to 0.321 BL (median: 0.031 BL; Figure 7E), confirming that the majority of confirmed NUMTs were phylogenetically proximate to their mitochondrial source sequences.

## 4 Discussion

### 4.1 Authentication Workflow and ASV Complexity

While discussed extensively in the context of metabarcoding (Alberdi et al. 2018; Andújar et al. 2021; Elbrecht, Peinert, and Leese 2017; Elbrecht, Vamos, et al. 2017; Lamb et al. 2019), the issue of extraneous sequences in deep amplicon sequencing from single specimens (‘megabarcoding’) has received less attention. Here, we show the challenges of implementing a filtering procedure to extract the true barcode haplotypes from a background of cross‐contamination and technical artefacts that can confound specimen identification and diversity estimates, but also highlight the potential of such data for studying species interactions, pseudogene variation and symbionts. At the core of our workflow are two authentication criteria: (i) minimum read count thresholds (MRCTs) and (ii) congruence of phylogenetic position and morphological identification. We selected a relatively low MRCT of four reads, which balanced high specimen retention (91.77%) with the removal of more than half of presumed artefactual ASVs. Each of the retained specimens yielded 9.5 ASVs on average, underlining the need for stringent filtering to recover the single authentic mitochondrial haplotype (Figure 2). Overall, the workflow extracted the presumed true mitochondrial haplotype for 86.52% of specimens (79.40% when PCR failures are considered).

While authenticated ASVs exhibited high mean read counts (1904 reads), the median was considerably lower (650 reads) and small‐bodied families (< 3 mm) achieved only 52.3% success compared to 88.9% for large‐bodied taxa (> 10 mm). A stringent abundance‐only threshold would disproportionately exclude these minute but ecologically significant lineages. The phylogenetic authentication framework thus ensures retention of low‐abundance authentic sequences from small or degraded specimens, while excluding high‐abundance contaminants, a distinction that read count alone cannot achieve.

### 4.2 ASV Recovery and Parameters of Authentication Success

Our categorisation of ASVs based on relative abundance, distance to known reference sequences and phylogenetic position revealed that technical artefacts constituted the largest failure class (102,083 records, 58.02%), dominated by low‐abundance variants with variable phylogenetic distances from members of the expected family (mean: 1.244). They illustrate the challenges of high‐throughput amplicon sequencing, especially when working with partially degraded field samples that increase PCR amplification artefacts, sequencing errors and sample contamination. Although many factors unique to each of the 14 sampling campaigns hampered strict standardisation, several trends aligned with expectations. Active collection methods achieved higher success rates than most passive methods, presumably because specimens were preserved immediately rather than remaining in traps for prolonged periods, thereby reducing exposure to environmental factors that accelerate DNA hydrolysis and oxidative damage (Marquina et al. 2019). Prolonged exposure in passive traps, particularly in tropical environments with high temperatures and humidity, promotes enzymatic degradation by endogenous nucleases, accelerates depurination and increases the likelihood of cross‐contamination from co‐trapped organisms through tissue dissolution and DNA leaching into the preservation fluid. The kind of preservation fluid also affects authentication success, with ethanol outperforming SDS + EDTA. Ethanol rapidly dehydrates tissues and inactivates nucleases, providing superior DNA preservation, whereas SDS + EDTA buffer, while effective at lysing cells and chelating divalent cations to inhibit DNases, may permit residual enzymatic activity at ambient tropical temperatures and promote DNA fragmentation during extended field exposure (Zizka et al. 2019). Trap type had an additional effect; for example, Malaise traps performed slightly better than flight interception traps, although these differences were overlain by the use of ethanol vs. SDS + EDTA in either trap type. Pitfall traps had the lowest success rate (71.2%), likely due to water ingress, soil‐derived PCR inhibitors (Schrader et al. 2012) and contamination from non‐target organisms. Finally, leaf litter sampling showed low scores, probably because of the inclusion of very small specimens, such as minute Ptiliidae that had a very low success rate, rather than any features associated with this trap type per se.

The latter highlights the strong effect of taxonomic identity as a predictor of success at the specimen level. Authentication success varied widely among families (0%–100%), reflecting differences in morphology, physiology and genome features. Families such as Chrysomelidae (93.0% success) likely yielded good DNA due to thinner cuticles or larger average body sizes, whereas heavily sclerotised or minute taxa performed poorly. The presence of metabolic compounds and defensive secretions may also affect DNA preservation and amplification. Thus, the wide range of authentication success across families suggests that extraction protocols may need to be optimised for particular morphological types. Geographic and batch effects were substantial (Section 3.5), highlighting the importance of standardised protocols.

From the ASV perspective, the probability that a given ASV is an authentic mitochondrial haplotype depends prominently on its phylogenetic position, as the correct family placement is a prerequisite for recognition as an authentic ASV. Thus, this parameter trivially has great predictive importance in the feature analysis, but other factors also contribute, notably read count and similarity to a sequence database entry, reflecting the fact that true mitochondrial copies are present in higher abundance (Shokralla et al. 2015). Conversely, the total ASV count per sample and the phylogenetic proximity to a mitogenome had little effect (Figure 6B). The mitogenomes used in the backbone tree were, in part, derived from the same specimens as the ASVs, but this does not appear to be relevant for the authentication pipeline, which uses the placement to family only. The current mitogenome tree comprising > 13,000 mitogenomes is presumably of sufficient quality and taxon density to allow for this family‐level placement, which is likely to be accurate for a wide range of species of Coleoptera. If used in other groups, the mitogenome tree may need to be established first, but the coverage of relevant sequences is growing rapidly (Cameron 2025), allowing the wider adoption of phylogeny‐based ASV authentication.

### 4.3 Classification of Secondary ASVs

Beyond the ‘authentic’ mitochondrial haplotype and technical artefacts, numerous secondary ASVs were recognised and classified by their phylogenetic position, read count and recurrence in multiple libraries. The largest group (24,977 ASV records, 14.20% of all records from accepted specimens) comprised ASVs with great phylogenetic distance to the primary ASV or morphological identification. Most of these (71.7%) represented cross‐contamination with beetle taxa appearing as primary ASVs in other samples. Contamination may have arisen either in the traps or during laboratory extraction and sequencing steps, but due to the combinations in which these specimens were processed, it was not possible to determine the dominant factor. The unique dual‐indexing strategy implemented here helps detect and correct index hopping; future studies may further reduce cross‐contamination through stricter laboratory protocols, including spatial separation of extraction and PCR steps and use of negative controls at each stage. The remaining ASVs in this category originated from non‐beetle arthropods (19.7%), probably due to trap by–catch, although some may reflect ecological interactions, such as prey items or parasitoids. Bacterial Rickettsiales (2.6%) were also detected, indicating potential endosymbionts.

A further 19,830 ASV records were closely related to a primary ASV (mean distance: 0.143) and likely represent NUMTs or heteroplasmic copies. Applying the co‐occurrence criterion, that is, the consistent presence of the same putative NUMT alongside the same authenticated anchor ASV in ≥ 2 independent specimens, identified 930 unique ‘confirmed’ NUMT sequences. Confirmed NUMTs were particularly frequent in Staphylinidae, Chrysomelidae and Scarabaeidae, which may reflect lineage‐specific NUMT abundance or the higher detection rate due to comparatively high numbers of sequence reads obtained for many specimens in these families. High NUMT diversity extends previous observations (Andújar et al. 2021; Song et al. 2008), and systematic co‐occurrence testing with authenticated anchor ASVs provides a rigorous, quantitative procedure for their validation. Confirmed NUMTs should be recorded alongside their authenticated anchor ASVs, as they are useful not only for filtering metabarcode data (Andújar et al. 2021) but also as potential markers for population genetics (Pons and Vogler 2005). Final confirmation requires genome sequencing (Hebert et al. 2025), but consistent co‐amplification with the primary mitochondrial haplotype makes confirmed NUMTs readily available as nuclear gene markers without requiring additional sequencing effort.

Sequence comparisons between the NUMT and anchor sets were used to obtain molecular evidence supporting the NUMT classification. The overall pattern of GC content and ENC is slightly shifted in the confirmed NUMTs compared to their matched anchor sequences (Figure 7A,B), but this did not translate into significant differences in within‐specimen LMM (all *p* > 0.05; Table S8). Thus these 930 confirmed NUMTs retain close compositional similarity to their mitochondrial source sequences. Yet, there were significant shifts in codon usage evident in RSCU (Figure 7C) and specifically in the frequency of 12 codons (Figure 7F, Table S7). The affected codons represent shifts towards GC as well as AT bases, showing that the nuclear copies do not primarily assume the lower AT content of the nuclear genome but additional drivers of sequence differentiation are effective in nuclear versus mitochondrial genomes. These subtle shifts, together with the co‐occurrence criterion, low intra‐individual abundance and phylogenetic congruence, collectively support the NUMT classification in this dataset. Although compositional similarity at the GC and ENC level is not a reliable discriminator between mitochondrial and nuclear copies in an individual case, the overall pattern and especially the shifts in codon usage, may reflect the onset of relaxed purifying selection following integration into the nuclear genomic environment (Bensasson et al. 2001; Hazkani‐Covo et al. 2010). These NUMTs span distances of 0.24%–9.81% from their presumed mitochondrial ancestors, but this distribution is highly right‐skewed (median 1.91%; 81.3% ≤ 5%; Figure 7D), presumably reflecting the decreasing chance of long‐term evolutionary survival of NUMTs (Pons and Vogler 2005), especially if they are pre‐filtered for reading frame errors and stop codons, as conducted here with the standard sequence filtering procedures (see Section 2). These findings also support the categorisation of the large number of other NUMTs not corroborated by the co‐occurrence analysis but whose classification was based on the same criteria.

A limitation of amplicon‐based classification is the difficulty of distinguishing NUMTs from heteroplasmic mitochondrial variants. Both produce secondary ASVs that consistently co‐amplify with a primary haplotype and are phylogenetically close to authenticated sequences and cannot be discriminated based on read abundance or phylogenetic placement criteria alone. Heteroplasmy is increasingly recognised as prevalent in insects, including in COI barcode data (Song et al. 2008; Prous et al. 2026), and a proportion of the 19,830 intra‐individual variant records, in particular those beyond the 930 co‐occurrence‐validated NUMTs, may represent heteroplasmic rather than nuclear copies. However, the codon usage analysis (Section 3.6) provides population‐level evidence consistent with nuclear origin for the confirmed NUMT subset, as the RSCU divergence documented here (Figure 7) reflects codon preference shifts consistent with relaxed purifying selection following nuclear genomic integration (Bensasson et al. 2001; Hazkani‐Covo et al. 2010). Such divergence would not be expected in heteroplasmic mitochondrial variants, which remain subject to mitochondrial selective pressures, which provides molecular support for the nuclear, rather than heteroplasmic, origin of the confirmed NUMT set at the population level. However, this aggregate evidence cannot resolve individual cases. Definitive discrimination between NUMTs and heteroplasmy will ultimately require long‐read or whole‐genome sequencing.

### 4.4 Implications for Biodiversity Assessment and Metabarcoding

Deep barcode sequencing of individual specimens remains underexplored, as most large‐scale studies either focus simply on top‐abundant reads (Hebert et al. 2025; Meier et al. 2016) or cluster the variation into OTUs (Hartop et al. 2024; Jabot et al. 2025; Yeo et al. 2021). Our results show that this practice overlooks the complexity of authentic, artefactual and secondary sequences, whose compositions differ systematically across taxa, preservation conditions, sequencing batches and others, which inevitably complicates reliable species detection. The implications of these findings for biodiversity studies are twofold: (i) The biases in read abundances and contamination observed in single‐specimen sequencing will inevitably be exacerbated in metabarcoding mixtures. When exposed to mixed templates, PCR preferentially amplifies well‐preserved, high‐concentration variants, obscuring other true variants and elevating certain contaminants instead. Our study helps predict which sequence types are likely to be under‐ or over‐represented and how this affects the diversity estimates across taxonomic, morphological and ecological groups. However, by using phylogenetic matching as a second criterion of validation, additional ASVs can be accepted if some information about the specimen mixture is available (e.g., images or reference sequences from related samples). (ii) Accurate ASV filtering is essential when using these sequences for expanding the reference sets of barcode data. The barcoding literature frequently laments the lack of adequate reference databases, especially in tropical invertebrate groups (e.g., Zinger et al. 2025). This gap can be reduced if validated high‐throughput barcodes are deposited in databases, rather than being used solely for identification and not reused, as is the current practice. However, these de novo‐generated reference data need to be accurate at the nucleotide level and need to be validated as authentic genomic templates. Additionally, it is crucial to maintain records of secondary reads that co‐amplify with the presumed true variant but are not removed by standard filtering methods, as they can impact species richness estimates and measures of genetic variation.

Treating deep‐sequenced amplicons as true genetic variants also links their use to phylogenetic inference and trait reconstruction. While we used phylogenetic analysis mainly for ASV authentication, the precise placement of these amplicons allows them to be integrated into annotated phylogenies, enabling trait imputation (Keck et al. 2018). We argue that full phylogenetic likelihood tree searches provide a more resolved and accurate phylogenetic position than standard ‘phylogenetic placement’ methods designed for metagenomics, which merely add query sequences to a fixed topology (Czech et al. 2022). This approach provides a preliminary evolutionary position for each species encountered, which, when combined with associated images and collecting data, contributes directly to a global, increasingly complete tree‐of‐life. We conclude that investment in reference tree construction provides greater long‐term benefits for biodiversity assessment from barcodes than procedural standardisation of data filtering alone.

The choice of sequencing platform significantly affects this authentication framework's reliance on exact ASV calling. While our deep Illumina MiSeq approach yielded 58.0% technical artefacts, 92.1% were low‐abundance reads (1–3 reads) effectively removed by implementing an abundance threshold based on high‐accuracy (> Q30) single‐nucleotide resolution (Callahan et al. 2017). Alternatively, Oxford Nanopore Technology (ONT) enables ultra‐high throughput (Hebert et al. 2025), but its higher‐error rates necessitate consensus‐based clustering rather than strict ASV‐level analysis. PacBio HiFi achieves single‐molecule, Sanger‐equivalent accuracy (Congrains et al. 2025) but remains less practical than Illumina for processing tens of thousands of specimens due to higher costs and lower throughput. Consequently, Illumina offers cost‐effective exact ASV resolution, PacBio HiFi provides premium reference‐quality haplotypes, and ONT maximises specimen throughput using OTUs. Although theoretically platform‐agnostic, our framework's phylogenetic placement step relies on the nucleotide‐level accuracy provided by Illumina or PacBio data and would require adaptation to handle ONT's persistent consensus errors.

### 4.5 Pipeline‐Agnostic Authentication: Conceptual Comparison With DADA2

The authentication framework developed here is designed to operate downstream of any denoising pipeline and is not restricted to the VSEARCH/UNOISE3 approach employed in the present study. A direct empirical comparison with DADA2 (Callahan et al. 2016) was not feasible, as the dataset comprises multiple independent sequencing projects with heterogeneous library preparation protocols, and DADA2 requires per‐run error model training from raw FASTQ files that were not uniformly available. Nonetheless, the key distinction between denoising and authentication is clear: both DADA2 and VSEARCH/UNOISE3 address technical sequence errors through quality filtering, denoising and chimaera removal, but neither can resolve biological contaminants that constitute genuine DNA sequences. In the present dataset, 57,970 records—comprising NUMTs (*n* = 19,830), environmental contamination (*n* = 24,977) and cross‐sample contamination (*n* = 13,163)—passed all denoising quality filters and were identified exclusively through specimen‐level authentication, phylogenetic placement and taxonomic congruence assessment (Figure S2; Table S6). This authentication gap is intrinsic to all read‐based denoising methods and cannot be addressed by adjusting error models or abundance thresholds alone. The authentication framework is therefore complementary to any denoising pipeline and could be applied equally downstream of DADA2 or VSEARCH/UNOISE3. Its pipeline‐agnostic design—requiring only the final ASV sequence table rather than raw reads—ensures that future improvements in denoising technology can be readily incorporated without modifications to the downstream authentication workflow.

## 5 Conclusions

By combining abundance‐ and phylogeny‐based filtering, we provide a stringent authentication framework that addresses shortcomings of existing approaches: unrecognised contamination within the traps or the laboratory, inflated species diversity estimates, unused information about ecological interactions and uncertainty of NUMT recognition. The systematic classification of ASVs by phylogenetic distance clarifies the biological and technical causes underlying complex amplicon variation. The framework also provides a robust approach to the identification of nuclear pseudogenes in (meta)barcoding, especially in cases of consistent co‐amplification with a major variant. The authenticated dataset, placed in a phylogenetic tree and underpinned with high‐resolution images and detailed collecting information, provides a valuable contribution to molecular taxonomic resources and demonstrates the feasibility of large‐scale single‐specimen authentication in taxonomically challenging tropical ecosystems. Not least, we are providing validated barcodes for nearly 13,000 species of mostly tropical beetles, of which only a small proportion has been sequenced previously. The approach represents a significant step towards evolutionary context and improved standards for large‐scale biodiversity assessment.

## Author Contributions

S.O. and A.P.V. designed research; S.O., H.L., Z.Z., M.P.C., T.J.C., C.A. and P.A. conducted fieldwork and specimen processing; S.O. and H.L. performed laboratory work; S.O., H.L., M.P.C., T.J.C. and Z.Z. performed bioinformatics analyses; S.O. analysed data; S.O. and A.P.V. wrote the paper with input from all authors. All authors reviewed and approved the final manuscript.

## Funding

This work was supported by the Institute for the Promotion of Teaching Science and Technology and Biodiversity Initiative of the Natural History Museum.

## Disclosure

Benefit‐Sharing Statement: The benefits of this research are shared through several mechanisms, in accordance with the principles of the Convention on Biological Diversity (CBD). First, through the establishment of international scientific collaboration, as reflected in the co‐authorship of this study. Second, all genetic data, analysis scripts and high‐resolution specimen images are made publicly available (see Data Availability Statement), ensuring results are shared with the broader scientific community, including researchers and institutions in the countries of origin. Finally, the research addresses a priority concern by developing a scalable framework for the biodiversity assessment of under‐characterised tropical fauna, which is essential for conservation efforts and sustainable resource management.

Permits: All specimens were collected in accordance with international Access and Benefit Sharing regulations. Details are available at the NHM Data Portal.

## Conflicts of Interest

The authors declare no conflicts of interest.

## Supporting information


**Figure S1:** Phylogenetic reference tree constructed from 13,380 complete or partial Coleoptera mitochondrial genome sequences used for ASV authentication. Tips represent individual mitogenomes; colours indicate family‐level taxonomic assignments.

**Figure S2:** (A) Comparison of DADA2 and VSEARCH/UNOISE3 denoising pipelines and their integration with the authentication framework. (B) ASV record composition at each processing stage showing reduction from all records (*n* = 175,954) to post‐MRCT threshold (*n* = 91,402) to fully authenticated ASVs (*n* = 15,901). (C) Capability comparison of DADA2, VSEARCH/UNOISE3 and the authentication framework across key analytical features.

**Table S1:** Authentication success rate by country of specimen collection. For each country, the table reports total specimen count, number of authenticated specimens, success rate, percentage and 95% confidence interval.

**Table S2:** Authentication success rate by specimen collection method. For each collection method, the table reports total specimen count, number of authenticated specimens, success rate, percentage and 95% confidence interval.

**Table S3:** Authentication success rate by sequencing project batch. For each project batch, the table reports total specimen count, number of authenticated specimens, success rate, percentage and 95% confidence interval.

**Table S4:** Authentication success rate by insect family. For each family, the table reports total specimen count, number of authenticated specimens, success rate, percentage and 95% confidence interval.

**Table S5:** Statistical test results for factors affecting COI authentication success. Includes chi‐square tests and linear mixed model results with effect sizes and interpretation for specimen‐level and family‐level factors.

**Table S6:** Comparative analysis of DADA2 denoising pipeline outputs versus the phylogenetic authentication framework used in this study, including pipeline feature comparison and ASV record composition at each processing stage.

**Table S7:** Relative synonymous codon usage (RSCU) comparison between authenticated ASVs and confirmed NUMTs for all 64 codons. Includes mean RSCU values, delta RSCU and Wilcoxon signed‐rank test results with Benjamini–Hochberg correction.

**Table S8:** Comparison of sequence quality and composition metrics between authenticated ASVs and NUMTs using cross‐specimen matched pairs. Includes Mann–Whitney *U* tests, effect sizes, linear mixed model coefficients and intraclass correlation coefficients.

**Data S1:** Tagged degenerate primer combinations used for COI amplicon generation, including Illumina adapter tail sequences, sample‐specific inline tags and degenerate primer sequences for all forward and reverse primer pairs used in this study.

**Data S2:** Detailed bioinformatics pipeline documentation for ASV authentication, including software dependencies and versions, step‐by‐step processing commands (trimming, merging, quality filtering, denoising, length filtering, translation validation, chimaera detection), quality control checkpoints, expected outputs and troubleshooting notes.

**Data S3:** Mitogenome reference sequences used for phylogenetic reference tree construction, including specimen identifiers, taxonomic classifications (species to class level), project sample identifiers, matched ASV identifiers, COI sequence similarity, amplicon length and protein‐coding gene annotations.

**Data S4:** Phylogenetic framework documentation for ASV authentication, including mitochondrial genome data collection and preparation, protein‐coding gene extraction, supermatrix construction, phylogenetic tree inference and quality assessment, software dependencies and expected output file structure.

## Data Availability

Sequence Data: Raw sequence reads, authenticated ASV sequences, specimen metadata and supplementary data are deposited in Dryad: https://doi.org/10.5061/dryad.v41ns1s9q. The dataset includes quality‐filtered ASV sequences (FASTA), ASV abundance tables, authentication classifications, specimen metadata (including collection localities, dates, trap types, preservation methods and morphological identifications), phylogenetic trees (Newick format), COI alignments and complete analysis outputs. Code Availability: The authentication pipeline and all analysis scripts for sequence processing, ASV authentication, phylogenetic placement and statistical analyses are available on GitHub: https://github.com/OunjaiS/ASV‐Selection‐Taxonomic‐Assignment and archived with a permanent DOI on Zenodo: https://doi.org/10.5281/zenodo.19538792. All third‐party scripts used in this study, including those from the tjcreedy/biotools repository (https://github.com/tjcreedy/biotools, accessed March 2023) and catfasta2phyml (https://github.com/nylander/catfasta2phyml), are documented with version information in Data S2 and S4. Specimen Images and Taxonomic Data: High‐resolution specimen images are archived on Flickr: https://www.flickr.com/photos/site‐100/. Taxonomic determinations were made to family or lower taxonomic level where possible, aided by expert taxonomists via iNaturalist: https://www.inaturalist.org/people/site_100.
