# Supplementary material for: Phylogenetic Authentication of Amplicon Sequence Variants in Single‐Specimen Metabarcoding of Tropical Insects
Source: Mol Ecol Resour. 2026 Jul 8;26(5):e70178. doi: 10.1111/1755-0998.70178 (PMC13346335; doi:10.1111/1755-0998.70178)
Supplement: Supplementary file 1 — Figure S1: Phylogenetic reference tree constructed from 13,380 complete or partial Coleoptera mitochondrial genome sequences used for ASV authentication. Tips represent individual mitogenomes; colours indicate family‐level taxonomic assignments. [file MEN-26-e70178-s011.pdf]

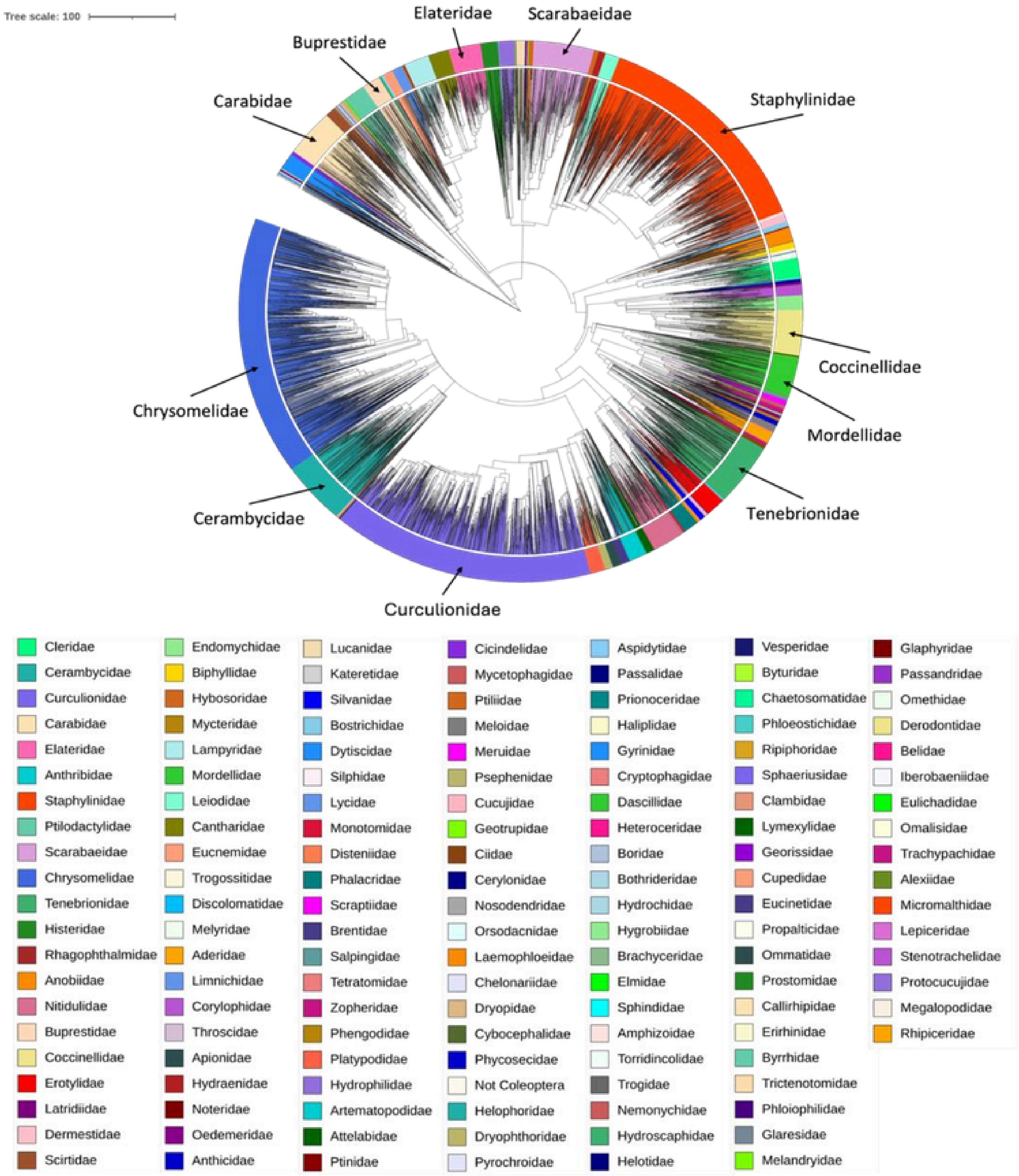
Tree scale: 100
Elateridae
Scarabaeidae
Buprestidae
Carabidae
Staphylinidae
Coccinellidae
Mordellidae
Tenebrionidae
Chrysomelidae
Cerambycidae
Curculionidae
Cleridae
Cerambycidae
Curculionidae
Carabidae
Elateridae
Anthribidae
Staphylinidae
Ptilodactylidae
Scarabaeidae
Chrysomelidae
Tenebrionidae
Histeridae
Rhagophthalmidae
Anobiidae
Nitidulidae
Buprestidae
Coccinellidae
Erotylidae
Latridiidae
Dermestidae
Scirtidae
Endomychidae
Biphyllidae
Hybosoridae
Mycteridae
Lampyridae
Mordellidae
Leiodidae
Cantharidae
Eucnemidae
Trogossitidae
Discolomatidae
Melyridae
Aderidae
Limnichidae
Corylophidae
Throscidae
Apionidae
Hydraenidae
Noteridae
Oedemeridae
Anthicidae
Lucanidae
Kateretidae
Silvanidae
Bostrichidae
Dytiscidae
Silphidae
Lycidae
Monotomidae
Disteniidae
Phalacridae
Scraptiidae
Brentidae
Salpingidae
Tetratomidae
Zopheridae
Phengodidae
Platypodidae
Hydrophilidae
Artematopodidae
Attelabidae
Ptinidae
Cicindelidae
Mycetophagidae
Ptiliidae
Meloidae
Meruidae
Psephenidae
Cucujidae
Geotrupidae
Ciidae
Cerylonidae
Nosodendridae
Orsodacnidae
Laemophloeidae
Chelonariidae
Dryopidae
Cybocephalidae
Phycosecidae
Not Coleoptera
Helophoridae
Dryophthoridae
Pyrochroidae
Aspidytidae
Passalidae
Prionoceridae
Haliplidae
Gyrinidae
Cryptophagidae
Dascillidae
Heteroceridae
Boridae
Bothrideridae
Hydrochidae
Hygrobiidae
Brachyceridae
Elmidae
Sphindidae
Amphizoidae
Torridincolidae
Trogidae
Nemonychidae
Hydroscaphidae
Helotidae
Vesperidae
Byturidae
Chaetosomatidae
Phloeostichidae
Ripiphoridae
Sphaeriusidae
Clambidae
Lymexylidae
Georissidae
Cupedidae
Eucinetidae
Propalticidae
Ommatidae
Prostomidae
Callirhipidae
Erirhinidae
Byrrhidae
Trictenotomidae
Phloiophilidae
Glaresidae
Melandryidae
Glaphyridae
Passandridae
Omethidae
Derodontidae
Belidae
Iberobaeniidae
Eulichadidae
Omalisidae
Trachypachidae
Alexiidae
Micromalthidae
Lepiceridae
Stenotrachelidae
Protocucujidae
Megalopodidae
Rhipiceridae
