# Supplementary material for: Phylogenetic Authentication of Amplicon Sequence Variants in Single‐Specimen Metabarcoding of Tropical Insects
Source: Mol Ecol Resour. 2026 Jul 8;26(5):e70178. doi: 10.1111/1755-0998.70178 (PMC13346335; doi:10.1111/1755-0998.70178)
Supplement: Supplementary file 2 — Figure S2: (A) Comparison of DADA2 and VSEARCH/UNOISE3 denoising pipelines and their integration with the authentication framework. (B) ASV record composition at each processing stage showing reduction from all records (n = 175,954) to post‐MRCT threshold (n = 91,402) to fully authenticated ASVs (n = 15,901). (C) Capability comparison of DADA2, VSEARCH/UNOISE3 and the authentication framework across key analytical features. [file MEN-26-e70178-s003.pdf]

(A) Denoising Pipelines and the Authentication Framework

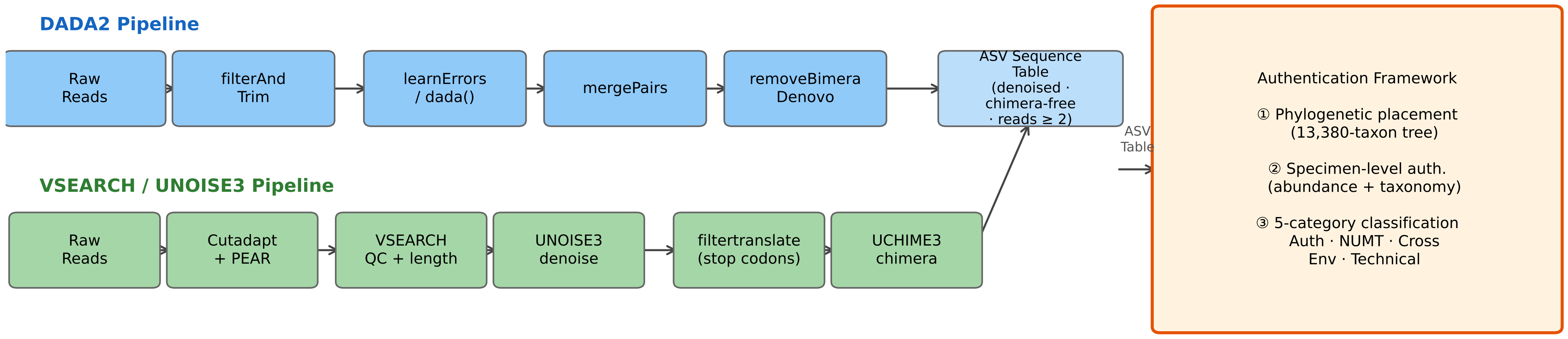

** Pipeline-agnostic: Authentication Framework applies downstream of either DADA2 or VSEARCH/UNOISE3*

(B) ASV Record Composition at Each Processing Stage

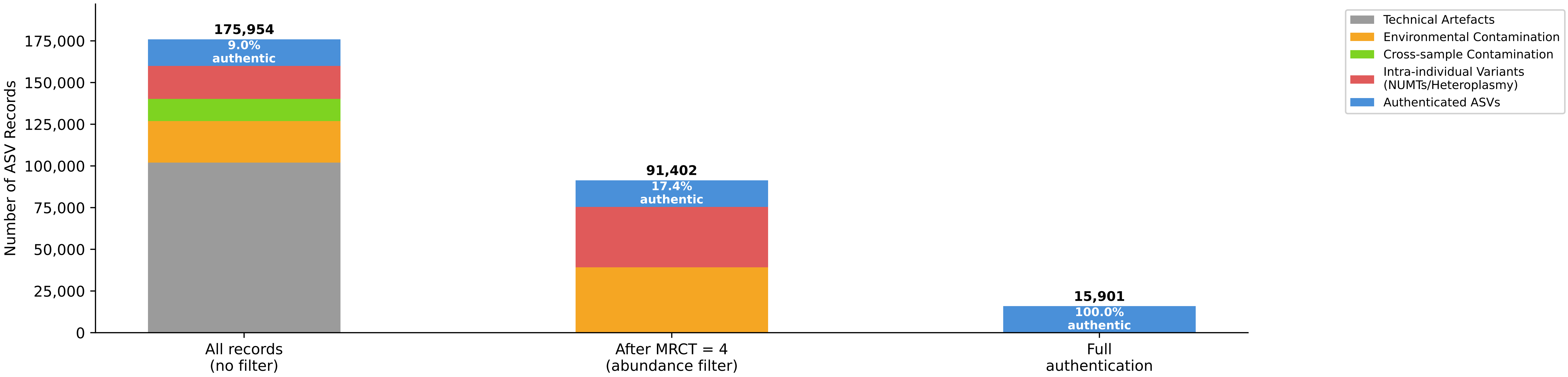

(C) Capability Comparison: DADA2 vs VSEARCH/UNOISE3 vs Authentication Framework

Capability	DADA2	VSEARCH/UNOISE3 + Authentication Framework
Denoising algorithm	DADA2 (parametric error model)	VSEARCH UNOISE3
Error model	+ parametric error model	**+ alpha-based (α = 2)**
Chimera detection	+ removeBimeraDenovo	**+ UCHIME3 de novo**
Min. abundance threshold	+ implicit (error model)	**+ MRCT = 4 reads**
Length filtering	+ optional (user-defined)	**+ 418 bp (COI amplicon)**
Translation / stop-codon filter	–	**+ invertebrate mitoch. code**
Specimen-level authentication	–	**+ abundance matching**
Phylogenetic placement	–	**+ 13,380-taxon reference tree**
Taxonomic congruence check	–	**+ 3-level family verification**
NUMT / heteroplasmy detection	–	**+ abundance + phylog. distance**
Cross-contamination detection	–	**+ specimen co-occurrence**
Environmental contamination detection	–	**+ taxonomic incongruence**
Intra-individual variant classification	–	**+ NUMT vs heteroplasmy class.**
Multi-category ASV output	– (binary: ASV or error)	**+ 5 categories**
ML-based confidence scoring	–	**+ ML confidence score**
