# Supplementary material for: Phylogenetic Authentication of Amplicon Sequence Variants in Single‐Specimen Metabarcoding of Tropical Insects
Source: Mol Ecol Resour. 2026 Jul 8;26(5):e70178. doi: 10.1111/1755-0998.70178 (PMC13346335; doi:10.1111/1755-0998.70178)
Supplement: Supplementary file 12 — Data S2: Detailed bioinformatics pipeline documentation for ASV authentication, including software dependencies and versions, step‐by‐step processing commands (trimming, merging, quality filtering, denoising, length filtering, translation validation, chimaera detection), quality control checkpoints, expected outputs and troubleshooting notes. [file MEN-26-e70178-s009.docx]

# Supplementary Material 2

# Detailed bioinformatics pipeline documentation for ASV authentication, including software dependencies and versions, step-by-step processing commands (trimming, merging, quality filtering, denoising, length filtering, translation validation, chimaera detection), quality control checkpoints, expected outputs, and troubleshooting notes.

# 1. Overview

This supplementary material provides a detailed step-by-step bioinformatics pipeline for processing raw metabarcoding sequence data through to authenticated Amplicon Sequence Variants (ASVs). The pipeline follows established best practices in metabarcoding analysis and integrates quality control measures at multiple stages to ensure data integrity. All commands and parameters are provided for reproducibility.

# 2. Software Requirements and Dependencies

## 2.1 Core Software

| **Software** | **Version** | **Purpose** |
| --- | --- | --- |
| Cutadapt | v3.5 | Primer trimming and adapter removal |
| PEAR | v0.9.6 | Paired-end read merging |
| VSEARCH | Latest | Quality filtering, dereplication, and chimaera detection |
| filtertranslate | Latest | Translation filtering (metaMATE package) |

## 2.2 Installation Commands

# Install Cutadapt
pip install cutadapt==3.5

# Install PEAR
# Download from https://cme.h-its.org/exelixis/web/software/pear/

# Install VSEARCH
# Download from https://github.com/torognes/vsearch

# Install metaMATE (for filtertranslate)
python3 -m pip install metaMATE

# 3. Step-by-Step Pipeline

## 3.1 Step 1: Primer Trimming and Adapter Removal

Objective: Remove primer sequences and adapters from demultiplexed paired-end reads whilst discarding sequences that do not contain the expected primer sequences.

**Input:** Demultiplexed paired-end FASTQ files (R1 and R2) in *0_demux/* directory

**Output:** Trimmed FASTQ files in *1_trimmed/* directory

Primer sequences:
Forward primer (-g): CCNGAYATRGCNTTYCCNCG
Reverse primer (-G): TANACYTCNGGRTGNCCRAARAAYCA

Commands:

# Create sample list
samples=$(for f in 0_demux/*.fastq; do s1=${f##*/}; echo ${s1%_*}; done | sort | uniq)
echo $samples

# Create output directory
mkdir 1_trimmed
rm -f 1_trim_report.txt

# Run trimming loop
for s in $samples
do
 cutadapt -j 20 \
 -g "CCNGAYATRGCNTTYCCNCG;min_overlap=5" \
 -G TANACYTCNGGRTGNCCRAARAAYCA \
 -o 1_trimmed/${s}_R1.fastq \
 -p 1_trimmed/${s}_R2.fastq \
 --minimum-length 50 \
 --discard-untrimmed \
 0_demux/${s}_R1.fastq \
 0_demux/${s}_R2.fastq \
 2>&1 | tee -a 1_trim_report.txt
done

Parameter justification:

| **Parameter** | **Value** | **Rationale** |
| --- | --- | --- |
| -j (threads) | 20 | Parallel processing for computational efficiency |
| min_overlap | 5 | Larger than default minimum overlap due to primer ambiguities (e.g., CCN = NCG) |
| --minimum-length | 50 bp | Removes very short reads that are likely sequencing errors |
| --discard-untrimmed | Yes | Removes reads without expected primers (potential contamination) |

Quality control check:

# Generate read count table
rm -f 1_trim_outcounts.tsv
for s in $samples
do
 for d in R1 R2
 do
 c=$(grep -c '^+$' 1_trimmed/${s}_$d.fastq)
 echo -e "$s\t$d\t$c" >> 1_trim_outcounts.tsv
 done
done

## 3.2 Step 2: Paired-End Read Merging

**Objective:** Merge overlapping paired-end reads into single sequences with quality score evaluation.

**Input:** Trimmed paired-end FASTQ files from *1_trimmed/*

**Output:** Merged single-end FASTQ files in *2_merged/* directory

Commands:

# Create sample list
samples=$(for f in 1_trimmed/*.fastq; do s1=${f##*/}; echo ${s1%_*}; done | sort | uniq)
echo $samples

# Create output directory
mkdir 2_merged
rm -f 2_merge_report.txt

# Run merging loop
for s in $samples
do
 pear -f 1_trimmed/${s}_R1.fastq \
 -r 1_trimmed/${s}_R2.fastq \
 -o 2_merged/$s \
 -q 26 \
 -v 60 \
 -j 20 \
 2>&1 | tee -a 2_merge_report.txt
done

# Clean up unneeded files
cd 2_merged/
rm *discarded* *unassembled*
rename -e "s/assembled\.//" *
cd ../

Parameter justification:

| **Parameter** | **Value** | **Rationale** |
| --- | --- | --- |
| -q (quality score) | 26 | Minimum quality score threshold for base calling accuracy |
| -v (minimum overlap) | 60 bp | Ensures sufficient overlap for confident merging |
| -j (threads) | 20 | Parallel processing for computational efficiency |

Quality control check:

# Generate read count table
rm -f 2_merge_outcounts.tsv
for s in $samples
do
 c=$(grep -c '^+$' 2_merged/$s.fastq)
 echo -e "$s\t$c" >> 2_merge_outcounts.tsv
done

## 3.3 Step 3: Sample Concatenation and Labelling

**Objective:** Concatenate all samples into a single file whilst preserving sample identity through header modification.

**Input:** Merged FASTQ files from *2_merged/*

**Output:** Single concatenated FASTQ file *3_mbc_concat.fastq*

Commands:

# Create sample list
samples=$(for f in 2_merged/*.fastq; do s1=${f##*/}; echo ${s1%.*}; done | sort | uniq)
echo $samples

# Identify sequence headers (first 10 characters)
head -q -n 1 0_demux/*.fastq | cut -c 1-10 | sort | uniq

# Concatenate files with sample labels
# Note: Replace @HEAD with actual header prefix from your data
rm -f 3_mbc_concat.fastq
for s in $samples
do
 sed -e "s/\(^@HEAD.*\) .*$/\1;sample=$s;/" 2_merged/$s.fastq >> 3_mbc_concat.fastq
done

**Important note:** The @HEAD placeholder in the sed command must be replaced with the actual sequence header prefix from your sequencing platform. Use at least 4 characters to avoid matching quality lines that begin with '@'.

## 3.4 Step 4: Quality Filtering

**Objective:** Filter sequences based on expected error rates and convert to FASTA format.

**Input:** Concatenated FASTQ file *3_mbc_concat.fastq*

**Output:** Quality-filtered FASTA file *3_mbc_concat.fasta*

Command:

vsearch --fastx_filter 3_mbc_concat.fastq \
 --fastq_maxee 1 \
 --fastaout 3_mbc_concat.fasta

**Parameter:** --fastq_maxee 1 sets the maximum expected error threshold to 1.0, discarding sequences with higher error probabilities.

## 3.5 Step 5: Dereplication

**Objective:** Collapse identical sequences whilst retaining abundance information.

**Input:** *3_mbc_concat.fasta*

**Output:** Dereplicated sequences *4_mbc_derep.fasta*

Command:

vsearch --derep_fulllength 3_mbc_concat.fasta \
 --output 4_mbc_derep.fasta \
 --sizeout \
 --relabel uniq

The --sizeout parameter annotates sequences with their abundance, whilst --relabel uniq provides systematic naming.

## 3.6 Step 6: Denoising

**Objective:** Remove sequencing errors and low-abundance sequences using the UNOISE algorithm.

**Input:** *4_mbc_derep.fasta*

**Output:** Denoised sequences *5_mbc_denoise.fasta*

Command:

vsearch --cluster_unoise 4_mbc_derep.fasta \
 --minsize 4 \
 --unoise_alpha 2 \
 --centroids 5_mbc_denoise.fasta

Parameter justification:

| **Parameter** | **Value** | **Rationale** |
| --- | --- | --- |
| --minsize | 4 | Minimum abundance threshold to distinguish genuine sequences from errors |
| --unoise_alpha | 2 | Sensitivity parameter for error correction algorithm |

## 3.7 Step 7: Length Filtering

**Objective:** Remove sequences with insertions or deletions by filtering to expected amplicon length.

**Input:** *5_mbc_denoise.fasta*

**Output:** Length-filtered sequences *6_mbc_indelfil.fasta*

Command:

vsearch --fastx_filter 5_mbc_denoise.fasta \
 --fastq_minlen 418 \
 --fastq_maxlen 418 \
 --fastaout 6_mbc_indelfil.fasta

**Length threshold:** 418 base pairs corresponds to the expected COI amplicon length for the primer set employed. Sequences deviating from this length likely contain indels and are removed.

## 3.8 Step 8: Translation Filtering

**Objective:** Remove sequences containing stop codons or frameshifts by validating translation to amino acids.

**Input:** *6_mbc_indelfil.fasta*

**Output:** Translation-validated sequences *7_mbc_transpass.fasta*

Command:

filtertranslate -i 6_mbc_indelfil.fasta \
 -t 5 \
 -o 7_mbc_transpass.fasta

**Parameter:** -t 5 specifies translation table 5 (invertebrate mitochondrial code), appropriate for Coleoptera COI sequences.

***Note:*** *If filtertranslate is not found, install metaMATE: python3 -m pip install metaMATE*

## 3.9 Step 9: Chimaera Detection and Removal

**Objective:** Identify and remove chimeric sequences using the UCHIME3 algorithm in de novo mode.

**Input:** *7_mbc_transpass.fasta*

**Output:** Final authenticated ASVs *8_mbc_final.fasta*

Command:

vsearch --uchime3_denovo 7_mbc_transpass.fasta \
 --nonchimeras 8_mbc_final.fasta

*The file* ***8_mbc_final.fasta*** *contains the final set of authenticated Amplicon Sequence Variants (ASVs) for downstream analysis.*

## 3.10 Step 10: Read Mapping to ASVs

**Objective:** Map all quality-filtered reads back to authenticated ASVs to generate abundance table.

**Input:** *3_mbc_concat.fasta (quality-filtered reads) and 8_mbc_final.fasta (ASVs)*

**Output:** ASV abundance table *reads_asv_map.tsv*

Command:

vsearch --search_exact 3_mbc_concat.fasta \
 -db 8_mbc_final.fasta \
 -otutabout reads_asv_map.tsv

The --search_exact parameter ensures only perfect matches are counted, maintaining the integrity of ASV assignments.

# 4. Quality Control Checkpoints

The pipeline incorporates multiple quality control checkpoints to ensure data integrity:

| **Pipeline Stage** | **Quality Control Measure** |
| --- | --- |
| 1. Trimming | Primer presence verification; minimum length threshold (50 bp) |
| 2. Merging | Minimum overlap (60 bp); quality score threshold (Q26) |
| 3. Quality filtering | Maximum expected error ≤1.0 |
| 4. Denoising | Minimum abundance (4 reads); UNOISE algorithm error correction |
| 5. Length filtering | Exact length match (418 bp) to remove indels |
| 6. Translation | Validation of reading frame; removal of sequences with stop codons |
| 7. Chimaera detection | UCHIME3 de novo chimaera identification and removal |

# 5. Expected Outputs and Data Tracking

## 5.1 File Structure

Upon completion, the following directory structure will be generated:

.
├── 0_demux/ # Input demultiplexed files
├── 1_trimmed/ # Primer-trimmed files
├── 2_merged/ # Merged paired-end reads
├── 3_mbc_concat.fastq # Concatenated all samples
├── 3_mbc_concat.fasta # Quality-filtered sequences
├── 4_mbc_derep.fasta # Dereplicated sequences
├── 5_mbc_denoise.fasta # Denoised sequences
├── 6_mbc_indelfil.fasta # Length-filtered sequences
├── 7_mbc_transpass.fasta # Translation-validated sequences
├── 8_mbc_final.fasta # Final ASVs (authenticated)
├── reads_asv_map.tsv # ASV abundance table
├── 1_trim_report.txt # Trimming statistics
├── 2_merge_report.txt # Merging statistics
├── 1_trim_outcounts.tsv # Read counts after trimming
└── 2_merge_outcounts.tsv # Read counts after merging

## 5.2 Read Count Tracking

Read retention should be monitored at each step to identify potential issues. Typical retention rates are:

| **Pipeline Stage** | **Expected Retention** | **Concerning If** |
| --- | --- | --- |
| Primer trimming | 85-95% | <75% |
| Paired-end merging | 80-90% | <70% |
| Quality filtering | 70-90% | <60% |
| Length filtering | 75-90% | <65% |
| Translation filtering | 85-95% | <75% |
| Chimaera removal | 90-98% | <85% |

# 6. Software Citations

Edgar, R.C. (2016) UCHIME2: improved chimera prediction for amplicon sequencing. BioRxiv, 074252.

Martin, M. (2011) Cutadapt removes adapter sequences from high-throughput sequencing reads. EMBnet.journal, 17, 10-12.

Rognes, T., Flouri, T., Nichols, B., Quince, C. & Mahé, F. (2016) VSEARCH: a versatile open source tool for metagenomics. PeerJ, 4, e2584.

Zhang, J., Kobert, K., Flouri, T. & Stamatakis, A. (2014) PEAR: a fast and accurate Illumina Paired-End reAd mergeR. Bioinformatics, 30, 614-620.

# 7. Troubleshooting Common Issues

## 7.1 Low Read Retention After Trimming

Potential causes:
• Incorrect primer sequences specified
• Primers already removed during demultiplexing
• High proportion of non-target amplification

Solutions:
• Verify primer sequences against laboratory records
• Check first 100 bp of raw sequences for primer presence
• Consider reducing min_overlap parameter if primers have ambiguities

## 7.2 Low Merging Success Rate

Potential causes:
• Insufficient read overlap (amplicon too long)
• Poor sequencing quality in overlap region
• Read length shorter than expected

Solutions:
• Check read length distribution in raw data
• Consider reducing minimum overlap (-v parameter)
• Assess base quality scores in overlap region

## 7.3 High Sequence Loss at Translation Filtering

Potential causes:
• Incorrect genetic code specified
• High proportion of pseudogenes (NUMTs)
• Frameshift errors from poor quality data

Solutions:
• Verify genetic code appropriate for target taxa
• Manually inspect failing sequences for patterns
• Consider stricter quality filtering in earlier steps

# 8. Additional Notes

8.1 Computational Resources
The pipeline is parallelised where possible using 20 threads (-j 20 parameter). Adjust this value based on available CPU cores. Typical processing time for a dataset of 10 million reads is approximately 2-4 hours on a 20-core system.

8.2 Reproducibility
All commands are deterministic and will produce identical results given the same input data. Random seeds are not required. Software version numbers should be recorded to ensure reproducibility.

8.3 Downstream Analysis
The final output file 8_mbc_final.fasta contains authenticated ASVs suitable for taxonomic assignment, phylogenetic analysis, and diversity assessments. The reads_asv_map.tsv file provides abundance data for community composition analyses.
