# Supplementary material for: Phylogenetic Authentication of Amplicon Sequence Variants in Single‐Specimen Metabarcoding of Tropical Insects
Source: Mol Ecol Resour. 2026 Jul 8;26(5):e70178. doi: 10.1111/1755-0998.70178 (PMC13346335; doi:10.1111/1755-0998.70178)
Supplement: Supplementary file 13 — Data S3: Mitogenome reference sequences used for phylogenetic reference tree construction, including specimen identifiers, taxonomic classifications (species to class level), project sample identifiers, matched ASV identifiers, COI sequence similarity, amplicon length and protein‐coding gene annotations. [file MEN-26-e70178-s008.docx]

# Supplementary Material 4

# Phylogenetic framework documentation for ASV authentication, including mitochondrial genome data collection and preparation, protein-coding gene extraction, supermatrix construction, phylogenetic tree inference and quality assessment, software dependencies, and expected output file structure.

# 1. Overview

This supplementary material provides a detailed protocol for constructing phylogenetic reference trees from mitochondrial genome sequences. The framework was designed to support phylogenetic placement of amplicon sequence variants (ASVs) for taxonomic authentication. Three reference tree variants were constructed with different taxon sampling densities (5,000, 12,000, and 13,380 mitogenomes) to assess the influence of taxon sampling on placement stability. All trees were inferred using consistent maximum likelihood methods to ensure that topological differences reflected taxon sampling rather than methodological variation.

# 2. Mitochondrial Genome Data Collection and Preparation

## 2.1 Sources of Mitochondrial Genome Sequences

Mitochondrial genome sequences were obtained from two primary sources:

**1. De novo assembled mitogenomes:**

Complete or partial mitochondrial genomes were assembled from shotgun sequencing data obtained from a subset of specimens included in this study. Assembly followed established reference-guided protocols (Creedy, 2024), utilising the NOVOPlasty assembler or equivalent tools. Quality control measures ensured that only high-quality assemblies (>90% completeness, no frameshifts or stop codons in protein-coding genes) were retained.

**2. GenBank mitochondrial genomes:**

Existing Coleoptera mitochondrial genome sequences were downloaded from the NCBI GenBank nucleotide database (accessed January 2023). Sequences were filtered to retain only those with complete or near-complete annotation of the 13 standard mitochondrial protein-coding genes (PCGs). This dataset ensured broad taxonomic coverage across Coleoptera.

## 2.2 Dataset Composition

The complete dataset comprised 13,380 mitochondrial genome sequences representing 119 beetle families. This extensive taxonomic sampling provided a comprehensive phylogenetic framework spanning the major lineages of Coleoptera. All mitochondrial genome sequences used in this study are provided in Supplementary Material 3.

# 3. Protein-Coding Gene Extraction and Processing

## 3.1 Gene Extraction from Mitogenomes

**Objective:** Extract the 13 standard mitochondrial protein-coding genes from GenBank-formatted mitochondrial genome files.

**Input:** *Mitochondrial genomes in GenBank format (mitogenomes.gb)*

**Output:** *Individual nucleotide FASTA files for each protein-coding gene in 1_nt_raw/ directory*

Command:

mkdir 1_nt_raw
extract_genes.py -g mitogenomes.gb \
 -o 1_nt_raw/ \
 -k \
 --genetypes CDS

The extract_genes.py script (from tjcreedy/biotools repository) parses GenBank annotation to extract coding sequences. The -k flag keeps original sequence identifiers, whilst --genetypes CDS restricts extraction to protein-coding genes only.

**Expected output files:**
ATP6.fa, ATP8.fa, COX1.fa, COX2.fa, COX3.fa, CYTB.fa, ND1.fa, ND2.fa, ND3.fa, ND4.fa, ND4L.fa, ND5.fa, ND6.fa

## 3.2 Translation to Amino Acid Sequences

**Objective:** Translate nucleotide sequences to amino acids using the invertebrate mitochondrial genetic code.

**Input:** *Nucleotide FASTA files from 1_nt_raw/*

**Output:** *Amino acid FASTA files in 2_aa_raw/ directory*

Commands:

mkdir 2_aa_raw

# Translate all genes in a loop
for file in 1_nt_raw/*
do
 translate.py 5 < $file > 2_aa_raw/${file#*/}
done

**Parameter:** The value '5' specifies NCBI genetic code table 5 (invertebrate mitochondrial code), which is appropriate for Coleoptera mitochondrial sequences. The translate.py script (from tjcreedy/biotools repository) handles translation with automatic stop codon removal.

## 3.3 Multiple Sequence Alignment

**Objective:** Align amino acid sequences for each protein-coding gene independently using translation-guided alignment.

**Input:** *Amino acid FASTA files from 2_aa_raw/*

**Output:** *Aligned amino acid sequences in 3_aa_aln/ directory*

Commands:

mkdir 3_aa_aln

# Align all genes using MAFFT with translation guidance
for file in 2_aa_raw/*
do
 mafft --globalpair \
 --maxiterate 1000 \
 --anysymbol \
 --thread 10 \
 $file > 3_aa_aln/${file#*/}
done

Parameter justification:

| **Parameter** | **Value** | **Rationale** |
| --- | --- | --- |
| --globalpair | Enabled | Uses global pairwise alignment algorithm for high accuracy |
| --maxiterate | 1000 | Maximum number of iterative refinement cycles for optimal alignment |
| --anysymbol | Enabled | Allows unusual amino acid symbols in sequences |
| --thread | 10 | Number of CPU threads for parallel processing |

## 3.4 Backtranslation to Nucleotide Alignments

**Objective:** Project amino acid alignments back to nucleotide space, preserving alignment positions whilst maintaining codon structure.

**Input:** *Aligned amino acid files (3_aa_aln/) and original nucleotide files (1_nt_raw/)*

**Output:** *Aligned nucleotide sequences in 4_nt_aln/ directory*

Commands:

mkdir 4_nt_aln

# Backtranslate all genes
for file in 3_aa_aln/*
do
 backtranslate.py -i $file \
 1_nt_raw/${file#*/} \
 5 > 4_nt_aln/${file#*/}
done

The backtranslate.py script (from tjcreedy/biotools repository) uses the amino acid alignment as a template and retrieves corresponding nucleotide triplets from the original unaligned nucleotide sequences. This approach ensures that alignment positions are maintained in nucleotide space, preserving phylogenetically informative indel patterns whilst allowing nucleotide-level substitution analysis.

# 4. Supermatrix Construction

## 4.1 Gene Concatenation

**Objective:** Concatenate individual gene alignments into a single supermatrix whilst recording partition boundaries for downstream phylogenetic analysis.

**Input:** *Aligned nucleotide files from 4_nt_aln/*

**Output:** *Concatenated supermatrix (5_nt_supermatrix.fasta) and partition file (5_nt_partitions.txt)*

Command:

catfasta2phyml.pl -c -fasta 4_nt_aln/* > 5_nt_supermatrix.fasta 2> 5_nt_partitions.txt

**Parameter:** -c flag enables concatenation mode, -fasta specifies input format. The script (from nylander/catfasta2phyml repository) concatenates alignments and outputs partition boundaries to stderr (redirected to 5_nt_partitions.txt).

Quality control check:

# Verify partition file
cat 5_nt_partitions.txt

Expected supermatrix length: Approximately 18,600 base pairs (13 protein-coding genes × ~1,400 bp average length).

# 5. Phylogenetic Tree Inference

## 5.1 Maximum Likelihood Tree Construction

**Objective:** Infer a maximum likelihood phylogenetic tree using FastTree with the GTR+CAT approximation model.

**Input:** *5_nt_supermatrix.fasta*

**Output:** *Phylogenetic tree in Newick format (reference_tree.nwk)*

Command:

FastTree -gtr -cat 20 -gamma -nt 5_nt_supermatrix.fasta > reference_tree.nwk

Parameter justification:

| **Parameter** | **Value** | **Rationale** |
| --- | --- | --- |
| -gtr | Enabled | Generalised time-reversible nucleotide substitution model |
| -cat | 20 categories | Site rate heterogeneity approximation using 20 rate categories |
| -gamma | Enabled | Gamma distribution of site rates after tree estimation |
| -nt | Nucleotide | Specifies nucleotide sequence data (as opposed to protein) |

**Model selection:** The GTR+CAT approximation was selected because it provides a computationally efficient alternative to GTR+Gamma for large datasets whilst maintaining comparable accuracy. FastTree has been extensively validated for phylogenetic inference with thousands of taxa and is particularly well-suited for mitochondrial genome datasets.

## 5.2 Construction of Reference Tree Variants

To assess the influence of taxon sampling density on phylogenetic placement stability, three reference tree variants were constructed from the same supermatrix alignment:

| **Tree Variant** | **Number of Taxa** | **Sampling Strategy** |
| --- | --- | --- |
| 5k tree | 5,000 | Taxa selected to maximise family-level diversity, ensuring all 119 beetle families were represented |
| 12k tree | 12,000 | Taxa selected to balance taxonomic and phylogenetic diversity, increasing within-family sampling |
| 13.3k tree | 13,380 | Complete dataset with all available mitogenomes |

Taxon subsampling procedure:

For the 5k tree:
• Families were ranked by species richness
• Taxa were selected proportionally to family diversity
• Minimum of 10 taxa per family was maintained where possible

For the 12k tree:
• Additional taxa were selected based on phylogenetic distance
• Within-family sampling was increased for species-rich families
• Geographic representation was considered where metadata were available

**Critical methodological control:** All three tree variants were inferred using identical FastTree parameters (GTR+CAT model with 20 rate categories). This ensures that any observed differences in phylogenetic placement stability reflect taxon sampling density rather than methodological variation.

# 6. Tree Quality Assessment and Validation

## 6.1 Taxonomic Consistency

The phylogenetic trees were evaluated for taxonomic consistency by examining whether recognised taxonomic groups (families, subfamilies, tribes) formed monophyletic clades. This assessment provides confidence that the phylogenetic framework captures established evolutionary relationships within Coleoptera.

## 6.2 Tree Structure and Representation

The final reference trees represented 119 beetle families spanning the major lineages of Coleoptera, including:

• Adephaga (ground beetles, tiger beetles, diving beetles)
• Polyphaga (the vast majority of beetle diversity)
 - Bostrichiformia (wood-boring beetles)
 - Chrysomeloidea (leaf beetles, longhorn beetles)
 - Cucujiformia (sap beetles, ladybirds, darkling beetles)
 - Curculionoidea (weevils, bark beetles)
 - Elateroidea (click beetles, soldier beetles, fireflies)
 - Scarabaeoidea (scarabs, stag beetles)
 - Staphylinoidea (rove beetles, carrion beetles)
 - Tenebrionoidea (darkling beetles, blister beetles)

# 7. Software Requirements and Dependencies

## 7.1 Core Software

| **Software** | **Version** | **Purpose** |
| --- | --- | --- |
| extract_genes.py | Latest | Gene extraction from GenBank files (tjcreedy/biotools) |
| translate.py | Latest | Nucleotide to amino acid translation (tjcreedy/biotools) |
| backtranslate.py | Latest | Amino acid alignment to nucleotide projection (tjcreedy/biotools) |
| MAFFT | v7.x | Multiple sequence alignment |
| catfasta2phyml.pl | Latest | Sequence concatenation (nylander/catfasta2phyml) |
| FastTree | v2.1.x | Maximum likelihood phylogenetic inference |

## 7.2 Installation Instructions

# Clone tjcreedy/biotools repository
git clone https://github.com/tjcreedy/biotools.git

# Download catfasta2phyml
wget https://raw.githubusercontent.com/nylander/catfasta2phyml/master/catfasta2phyml.pl
chmod +x catfasta2phyml.pl

# Install MAFFT
sudo apt-get install mafft
# Or download from https://mafft.cbrc.jp/alignment/software/

# Install FastTree
sudo apt-get install fasttree
# Or download from http://www.microbesonline.org/fasttree/

# 8. Complete Workflow Summary

The complete phylogenetic framework construction workflow consists of the following sequential steps:

| **Step** | **Process** | **Input** | **Output** |
| --- | --- | --- | --- |
| 1 | Gene extraction | Mitogenomes (GenBank) | 13 gene files (nucleotide) |
| 2 | Translation | Nucleotide sequences | Amino acid sequences |
| 3 | Alignment | Amino acid sequences | Aligned amino acid sequences |
| 4 | Backtranslation | AA alignment + NT sequences | Aligned nucleotide sequences |
| 5 | Concatenation | 13 aligned gene files | Supermatrix (18,600 bp) |
| 6 | Tree inference | Supermatrix | Phylogenetic tree (Newick) |
| 7 | Tree variants | Subsampled supermatrix | 5k, 12k, 13.3k trees |

# 9. Expected Outputs and File Structure

Upon completion, the following directory structure will be generated:

├── mitogenomes.gb # Input GenBank file
├── 1_nt_raw/ # Extracted nucleotide genes (13 files)
│ ├── ATP6.fa
│ ├── ATP8.fa
│ ├── COX1.fa
│ └── ... (10 more genes)
├── 2_aa_raw/ # Translated amino acid sequences
├── 3_aa_aln/ # Aligned amino acid sequences
├── 4_nt_aln/ # Backtranslated nucleotide alignments
├── 5_nt_supermatrix.fasta # Concatenated supermatrix (~18,600 bp)
├── 5_nt_partitions.txt # Partition boundaries
├── reference_tree_13.3k.nwk # Complete tree (13,380 taxa)
├── reference_tree_12k.nwk # 12k tree variant
└── reference_tree_5k.nwk # 5k tree variant

**Total estimated processing time:** Approximately 10-18 hours for the complete 13.3k tree on a standard workstation (10-core CPU, 32 GB RAM). The 5k and 12k tree variants require proportionally less time.

# 10. Software Citations

Creedy, T.J. (2024) Reference-guided mitochondrial genome assembly protocols. bioRxiv.

Katoh, K. & Standley, D.M. (2013) MAFFT multiple sequence alignment software version 7: improvements in performance and usability. Molecular Biology and Evolution, 30, 772-780.

Price, M.N., Dehal, P.S. & Arkin, A.P. (2010) FastTree 2 – approximately maximum-likelihood trees for large alignments. PLoS ONE, 5, e9490.

# 11. Additional Notes and Best Practices

## 11.1 Data Quality Standards

Before inclusion in the phylogenetic framework, mitochondrial genomes should meet the following quality criteria:

• Completeness: ≥90% of the 13 protein-coding genes annotated
• Reading frames: No premature stop codons or frameshifts in PCGs
• Sequence quality: No ambiguous nucleotides (Ns) in coding regions
• Taxonomic verification: Species identification confirmed through morphology or DNA barcoding

## 11.2 Backbone Constraint Usage

The resulting phylogenetic trees serve as backbone constraints for downstream phylogenetic placement of ASV sequences. This approach ensures that barcode sequences are placed within a well-resolved phylogenetic framework whilst avoiding the computational burden of full phylogenetic inference with thousands of additional short sequences.

## 11.3 Tree Variant Selection

The three tree variants (5k, 12k, 13.3k) allow assessment of placement stability across different levels of taxonomic sampling. In practice:

• The 5k tree provides fastest computational performance for initial analyses
• The 13.3k tree offers maximum phylogenetic resolution for final authentication
• Consistent placement across all three trees provides high confidence in taxonomic assignments

## 11.4 Reproducibility Considerations

All commands in this protocol are deterministic and will produce identical results given the same input data. Software version numbers should be recorded to ensure complete reproducibility. All input mitochondrial genome sequences are provided in Supplementary Material 2, enabling full replication of the phylogenetic framework.
